# Separation and identification of bioactive peptides from stem of *Tinospora cordifolia* (Willd.) Miers

**DOI:** 10.1371/journal.pone.0193717

**Published:** 2018-03-01

**Authors:** Raman Pachaiappan, Ekant Tamboli, Aurovind Acharya, Chia-Hung Su, Subash C. B. Gopinath, Yeng Chen, Palaniyandi Velusamy

**Affiliations:** 1 Department of Biotechnology, School of Bioengineering, SRM University, Kattankulathur, Tamil Nadu, India; 2 Department of Chemical Engineering, Ming Chi University of Technology, Taishan, Taipei, Taiwan; 3 School of Bioprocess Engineering, Arau, Universiti Malaysia Perlis, Perlis, Malaysia; 4 Institute of Nano Electronic Engineering, Kangar, Universiti Malaysia Perlis, Perlis, Malaysia; 5 Department of Oral & Craniofacial Sciences, Faculty of Dentistry, University of Malaya, Kuala Lumpur, Malaysia; 6 Oral Cancer Research & Coordinating Center (OCRCC), Faculty of Dentistry, University of Malaya, Kuala Lumpur, Malaysia; INRA, FRANCE

## Abstract

Enzyme hydrolysates (trypsin, papain, pepsin, α-chymotrypsin, and pepsin-pancreatin) of *Tinospora cordifolia* stem proteins were analyzed for antioxidant efficacy by measuring (1) 1,1-diphenyl-2-picrylhydrazyl (DPPH^•^) radical scavenging activity, (2) 2,20-azinobis(3-ethylbenzothiazoline-6-sulfonic acid) (ABTS^**+**^) radical scavenging capacity, and (3) Fe^2+^ chelation. Trypsin hydrolysate showed the strongest DPPH^•^ scavenging, while α-chymotrypsin hydrolysate exhibited the highest ABTS^**+**^ scavenging and Fe^2+^ chelation. Undigested protein strongly inhibited the gastrointestinal enzymes, trypsin (50% inhibition at enzyme/substrate ratio = 1:6.9) and α-chymotrypsin (50% inhibition at enzyme/substrate ratio = 1:1.82), indicating the prolonged antioxidant effect after ingestion. Furthermore, gel filtration purified peptide fractions of papain hydrolysates exhibited a significantly higher ABTS^+^ and superoxide radical scavenging as compared to non-purified digests. Active fraction 9 showing the highest radical scavenging ability was further purified and confirmed by MALDI-TOF MS followed by MS/MS with probable dominant peptide sequences identified are VLYSTPVKMWEPGR, VITVVATAGSETMR, and HIGININSR. The obtained results revealed that free radical scavenging capacity of papain hydrolysates might be related to its consistently low molecular weight hydrophobic peptides.

## Introduction

Reactive oxygen species (ROS) are described as chemically reactive molecules having oxygen radicals and non-radicals that function as strong oxidants and the potential of easier conversion to free radicals. Some candidates are hydroxyl radical, hydrogen peroxide, superoxide anion, and hypochlorite ion. ROS is a by-product of normal mitochondrial oxidative metabolism and also a consequence of cellular response to bacterial pathogenesis, environmental xenobiotics and cytokines [1]. ROS is important mediators of redox cell signaling and the drivers of important regulatory metabolic pathways [2]. The natural defense systems to mitigate the effects of ROS comprise the superoxide dismutase enzymes that reduce the superoxide anion to hydrogen peroxide, catalase, and glutathione peroxidase that neutralize the effects of hydrogen peroxide. In addition, metal binding proteins like ferritin, metallothionein, ceruloplasmin, transferrin, and albumin bind transition metal ions (e.g. Fe^2+^) to nullify the metal ion-mediated free radical generation [1].

Oxidative stress is the consequence of non-bioavailability of the *in vivo* antioxidant defense molecules to scavenge the ROS completely, which lead to an increased production of ROS. This could be due to aging, unhealthy lifestyle habits (tobacco consumption), and exposure to environmental pollutants and excessive UV radiation [3,4]. Oxidative damage of major cellular biomolecules like lipids, proteins, DNA and carbohydrates is one of the main consequences of the disruption in antioxidant-pro-oxidant balance [5]. This may induce several abnormalities like chronic inflammation, atherosclerosis, lipid peroxidation, and carcinogenesis [1]. To ameliorate oxidative stress and improve the adequacy of free radical scavengers *in vivo*, the role of preventive and supportive therapy against oxidative stress becomes important. This can be provided in the form of dietary foods and products of plant origin, which has high antioxidant properties with a level of safety and efficacy not available in synthetic molecules.

However, the discovery of the role of proteins and amino acids as important components of the redox homeostasis maintenance system in cell metabolism has ignited interest in research on the role of protein and protein hydrolysates as potential antioxidants [6]. One of the major sources of bioactive proteins and peptides are foods; and they are naturally produced during fermentation or digestion in the gastrointestinal tract [7–9]. Proteins and bioactive peptides are produced by limited enzymatic hydrolysis from soybean, whey, maize, zein, buckwheat, and potato which are reported to have antioxidant, anti-inflammatory, anticancer, and other beneficial bioactivities [10–12]. Intracellular and extracellular proteolytic enzymes can be used effectively to produce bioactive peptides [13]. Antioxidative peptides, in general, have become a prominent research of interest owing to their simpler structure, high activity, and low immunoreactivity. The same applies for low molecular weight antioxidant proteins. Though numerous primary metabolites isolated from agricultural crops and milk products, are found to have free radical scavenging and metal chelation abilities, very less research has revealed on the antioxidant potential of proteins and peptides derived from medicinal plants, with more focus on secondary metabolites like flavonoids, carotenoids, α-tocopherol and other compounds.

In Indian medicinal system, *Tinospora cordifolia* plant is widely used due to its antioxidant, antidiabetic, anti-inflammatory, hepatoprotective, immunomodulatory, anti-neoplastic, and antimicrobial effects [14–16]. So far, these properties are identified from the secondary metabolites or aqueous extracts of *T*. *cordifolia* plant, wherein the active compound for specific biological activities may or may not be determined [17–19]. For example, ethanolic extracts of all parts of *T*. *cordifolia* showed hepatoprotective activity against carbon tetrachloride-induced hepatic damage in rats [20], but the bioactive components were not identified. Similarly, immunomodulatory properties of three species of Tinospora, in the form of guduchi-satwa, a well-known dosage form have been established [21]. Thus, this study aims to identify whether *T*. *cordifolia* proteins and peptides obtained after enzymatic hydrolysis have antioxidant activity.

## Methods

### Materials

*Tinospora cordifolia* stems were collected and their proteins were isolated. Enzymes were purchased from Sigma and Calbiochem, Merck. Pepsin was from porcine gastric mucosa (Calbiochem, Merck, activity: 3000 U/mg of protein calculated based on the substrate hemoglobin), Porcine pancreatic trypsin (Sigma, 10,000 U/mg of protein using the substrates benzoyl arginine ethyl ester, BAEE), bovine pancreatic α-chymotrypsin (Sigma, 40 U/mg of protein using the substrates benzoyl tyrosine ethyl ester, BTEE), porcine pancreatin (Sigma), and Soybean trypsin inhibitor (Calbiochem, Merck). The reagents required for the antioxidant assays were: 1,1-diphenyl-2-picrylhydrazyl(DPPH) (Sigma, minimum 95% purity by TLC), 2,2’-azinobis(3-ethylbenzothiazoline-6-sulfonic acid) diammonium salt (ABTS) (HPLC grade, Sigma), Ferrozine (extrapure, SRL) Ferrous chloride (extrapure, SRL), and Pyrogallol (HPLC grade, Sigma).

### Identification of plant

The experimental plant has been systematically characterized as *Tinospora cordifolia* by local flora with the voucher specimen No.: B0223 at School of Bioengineering, SRM University, Chennai, India. The molecular characterization of the plant has also been done with genomic DNA isolation using matK gene. The obtained sequence was then subjected to a BLAST search of the GenBank database (NCBI, Bethesda, MD, USA) to identify the experiment plant. Alignment of the sequence plant with the similar sequences in the GenBank database revealed a high similarity (99%) to *Tinospora cordifolia*. Based on these results, the experimental plant was identified as a member of *Tinospora cordifolia*, and the sequence has been deposited in GenBank (NCBI) with Accession No: MF459677.

### Extraction of *T*. *cordifolia* stem proteins

*T*. *cordifolia* plant stems were collected from SRM University campus, Kattankulathur, India. Extraction of proteins was performed according to the method described by Aranha et al. [22] with minor changes. The stems were washed with distilled water, rinsed with deionized Millipore water (18.2 mΩ), chopped into small pieces and air-dried at room temperature for 15 days to remove the residual moisture. The dried stems were then ground into fine dry powder using a mixer-grinder while maintaining at a temperature of 4°C and then immediately stored at -20°C for further processing. The powder was then re-suspended in deionized water at powder:liquid ratio of 1:5 (wt/vol). After stirring at 4°C (for 2 h), the suspension was centrifuged (5000 g) for 20 min. The supernatant was subjected to salt precipitation by slowly dissolving ammonium sulfate by stirring to 80% saturation. The resulting suspension was allowed to stand overnight at 4°C then centrifuged at 5000 g for 20 minutes. The precipitated proteins were re-suspended in a minimum amount of water and dialyzed using 3.5 kDa cut-off dialysis membrane (Thermo Scientific Snake Skin dialysis tubing, 3.5K MWCO, 16 mm ID) for 8 hours against Millipore (deionized) water. The dialyzed proteins were lyophilized (Lyodel Freeze Dryer Model: DPRG-1GH) to get dry powder which was aliquoted and stored at -20°C. The lyophilized powder was used for further experiments and assays. Proteins quantification was done by Bradford method [23].

### Preparation of *in vitro T*. *cordifolia* stem protein digests

A variety of fluids containing single or multiple enzymes were prepared to simulate the digestive activities of different regions of the gastrointestinal system *in vitro*. Both plant and animal digestive enzymes were used to determine the antioxidative efficacy of different enzyme digests (S1 Table).

To simulate *in vitro* gastric digestion, papain and pepsin enzymes were individually used for hydrolysis of the proteins. For papain digestion, 5 mg lyophilized powder of *T*. *cordifolia* stem extract was dissolved in 1ml of phosphate buffer (50 mM; pH 6.8). Papain activation solution contained 4 mg papain enzyme, 20 mM cysteine, and 50 mM EDTA in 20 ml pH 6.8 phosphate buffer [24,25]. 250 μl of papain solution (0.05 mg enzyme) was treated with a protein solution to give an enzyme/substrate ratio of 1:100, and 1500 U papain per mg proteins in the final reaction mixture. The reaction control consisted of proteins in buffer without the enzyme (1 ml, 5 mg/ml). The digestion mixture was incubated for digestion times of 30, 60, 80, 100, and 120 min in an orbital shaker (120 rpm, 37 °C) and 250 μl aliquots were withdrawn. The reaction was stopped by boiling the reaction mixture for six minutes to irreversibly denature papain enzyme. For pepsin digestion, suspensions of the *T*. *cordifolia* lyophilized powder were prepared in simulated gastric fluid (SGF)– 10 mM HCl, 30 mM NaCl, and pH 2.2, at 10mg/ml. After completion of incubation (at 37 °C) for 10 min, pepsin (2000 U/mg, gastric buffer pH 2.2, 0.5 mg/ml) was added (6 ml) to yield 6000 U of pepsin/ mg of protein as a final digestion mix containing 10 mg of protein and 3.33 mg of pepsin (pepsin/ protein ratio of 1:3.33, w:w). The reaction control is comprised of proteins in SGF (0.5 ml, 10 mg/ml) without the enzyme. Samples were incubated in a shaking incubator (120 rpm, 37 °C) and aliquots (500 μl) collected after 60 and 120 min. Digestions were terminated by increasing the pH to 8 by adding 160mM of sodium carbonate (45 μl) to irreversibly inactivate pepsin [26,27].

For the simulation of *in vitro* intestinal digestion, trypsin and α-chymotrypsin enzymes were separately used for hydrolysis of the proteins. The suspension of lyophilized *T*. *cordifolia* stem protein extract was made in Tris-HCl buffer having pH 7.8 and 8.0, respectively, adjusted with 1 M HCl to yield a final concentration, 5 mg/ml. Solutions of enzymes were added to the protein solutions in buffer to give the following: 1) For α-chymotrypsin digestion—Tris-HCl buffer pH 7.8, 8 U bovine α -chymotrypsin per mg of protein extract; 2) For trypsin digestion—Tris-HCl buffer pH 8.0, 2000 U of porcine trypsin per mg of test protein extract [28,29]. This yielded an enzyme/substrate ratio of 1:5 (w/w) for both trypsin and chymotrypsin (i.e. 5 mg of protein incubated with 1 mg of trypsin and chymotrypsin respectively). 1 ml of protein solution (5 mg/ml) was incubated as reaction control without enzyme addition. Digestions were carried out in a shaking incubator (120 rpm, 37 °C) and aliquots (100 μl) taken out at 30, 60, 120, and 180 min for further analysis. Reactions were stopped by boiling at 100 °C for 6 min to irreversibly denature the α-chymotrypsin and trypsin in the digestion mixes.

Apart from single enzyme digestion, the digestibility of *T*. *cordifolia* proteins was checked in a simulated gastrointestinal fluid comprising of all major gastric and pancreatic enzymes. The lyophilized protein powder was exposed to the first step of digestion by incubation in SGF– 10 mM HCl and 30 mM NaCl, pH 2.2, and pepsin (0.33% w/v, E/S ratio of 1:3.33). Incubation in SGF was performed with the following specifications: 666.67 U pepsin per mg of protein and was maintained in a shaking incubator (120 rpm, 37°C). Two hours of digestion in SGF was quenched by an addition of 40 μl of 0.9 M NaHCO_3_ per ml of digestion mixture to raise the pH to 7.5 followed by an addition of pancreatin (E/S ratio of 1:20). The reaction was continued for 4 hours with the same condition as for pepsin followed by boiling for 6 min to inactivate all enzymes.

### SDS-PAGE analysis

Tricine-SDS–PAGE with the protein digests were performed by the Schagger (2006) method [30,31]. Samples were taken at different time-points during *in vitro* gastric and duodenal digestion was kept at -20 °C until SDS–PAGE analysis. Briefly, 20 μg (different sample volumes for different digests) of the digested protein aliquots (for all enzymes), undigested protein, and enzyme (reaction mixture equivalent, i.e. 0.2 μg of papain, 6.67 μg of pepsin and 4 μg of trypsin and chymotrypsin respectively) was incubated with equal volume of 1X Tricine-SDS-PAGE reducing loading dye for 15 min at 45 °C before being loaded onto the gel along with lower range Genei protein markers (6 μl) for comparison. Protein bands were visualized by silver staining method. BIORAD ChemiDoc XRS+ imaging system was used for taking the images of the gels.

### Effect on intestinal enzyme activity

The effect of the *T*. *cordifolia* proteins on the hydrolyzing capacity of the intestinal enzymes, trypsin, and α-chymotrypsin was analyzed both qualitatively and quantitatively using disc diffusion and spectrophotometric assays, respectively. Gelatin-agar plates were made by adding 2.3 g of agar, 0.4% (w/v) gelatin to 100 mL of Tris-HCl buffer (10 mM; pH 8.0 for trypsin and pH 7.8 for α-chymotrypsin), and the solution was then autoclaved. The mixture was transferred into sterile Petri dishes under the sterile condition and solidified. A mixture of protein solutions at different concentrations and both trypsin and α-chymotrypsin enzymes (5 μl, 1 μg/μl; i.e. 50 U of trypsin and 0.2 U of α-chymotrypsin) in Tris-HCl buffer (pH 8.0) were pre-incubated for 1 hour and then pipetted into the respective wells. Both enzyme suspensions in buffer only were used as positive controls and Tris-HCl buffer alone was used as a negative control. For comparison of the efficacy of trypsin enzyme inhibitory action, a standard soybean trypsin inhibitor was also loaded into a new experimental gelatin plate along with all the other reaction mixtures as mentioned above for trypsin. The plates were incubated for 20 hours at room temperature. The gelatin plates were then stained using Coomassie Brilliant Blue R-250 (0.5%) staining reagent for 12 hours followed by destaining with water where the observations were recorded.

For the quantitative analysis of the effect of *T*. *cordifolia* stem proteins on both trypsin and α-chymotrypsin enzymes, spectroscopic analysis at 640 nm by Sigma’s non-specific protease assay using casein as a substrate and Folin-ciocalteu’s phenol reagent (FCP) (2N, Sigma-Aldrich) was adopted [32]. 500 μg trypsin enzyme solution (1 μg/μl, i.e. 5000 U; 10 mM sodium acetate buffer with 5 mM Calcium acetate; pH 8.0) was incubated with different amounts of lyophilized stem extract (50 μl sample volume) for 60 min. After pre-incubation, 5 ml of 0.65% casein in 50 mM Potassium phosphate buffer (pH 7.5) was added as a substrate and incubated at 37°C for 60 min. the reaction was terminated by adding 110 mM cold Trichloroacetic acid (TCA; 5 ml) followed by 30 minutes incubation. The reaction mixture was centrifuged and the absorbance of the filtrate was observed at 640 nm after adding 5 ml Na_2_CO_3_ and 1 ml of FCP. 50 μl of sodium acetate buffer was considered as the assay blank while 500 μg trypsin enzyme solutions were considered as a positive control. The conditions mentioned above were also maintained for α-chymotrypsin (1 μg/μl, i.e. 20 U) with a slight variation of pH of the sodium acetate buffer maintained at 7.8 to ensure optimum enzyme activity. The percentage of inhibition of the enzyme was estimated using the formula:

$$\%\;Inhibition\;=\;\frac{Absorbance\;of\;Blank-Absorbance\;ofSample}{Absorbance\;of\;blank}\times 100$$

### Determination of radical scavenging activity

The antioxidant properties of *T*. *cordifolia* proteins, protein hydrolysates, and purified protein/peptide fractions were analyzed by quantifying three high-throughput radical scavenging assays which are DPPH (Diphenyl Picryl hydrazine) radical scavenging assay, ABTS (2,2’-azinobis(3-ethylbenzothiazoline-6-sulfonic acid) diammonium salt) radical scavenging assay and the superoxide assay. While DPPH^•^ and ABTS^•+^ scavenging assays were performed for the different protein digests, ABTS^•+^ and superoxide (O_2_^•-^) assays were executed for the purified fractions of the papain digests.

For DPPH radical scavenging assay, we followed the Brand-Williams et al. procedure [33] was modified and performed in 96-well plates while 0.625 μM DPPH was dissolved in HPLC-grade methanol to generate DPPH^•^ free radicals. 20 μl aliquots of aqueous protein extracts at various concentrations (from 3 mg/ml to 0.5 mg/ml), and protein digests (2 mg/ml; digestion aliquots of 30, 60, 120, and 180 minutes) were added with 190 μl of DPPH solution diluted in methanol (160 μM, absorbance = 1 ± 0.05) and loaded onto a 96-well microplate. The mixture was shaken for a few seconds and incubated in the dark for 16 hours at room temperature. The absorbance was read at 517 nm in a microplate reader (Multiscan GO, Thermo Scientific). The radical scavenging activity of the samples was calculated as the percentage reduction of DPPH^•^ according to the following formula:

$$\%\;Reduction\;=\;\frac{Absorbance\;of\;blank-Absorbance\;of\;sample}{Absorbance\;of\;blank}\times 100$$

DPPH^•^ scavenging activity of the samples was compared with the standard antioxidant that is ascorbic acid (3 mg/ml to 0.5 mg/ml) [10].

The ABTS^•+^ scavenging ability was estimated by following the decolorization assay by using the method of Re et al. [34] with additional modifications. An aqueous solution of 7 mM of ABTS and 2.45 mM of potassium persulphate was made and incubated in the dark (for 12–16 h) to generate ABTS^•+^. The resulting dense-green colored solution was diluted with water prior to the assay to give an absorbance of 0.7 ± 0.02 at 734 nm. The RSA was then measured by using 10 μl samples (different concentrations of protein;2 mg/ml, protein digestion aliquots of 0, 30, 60, 90, 120, and 180 minutes, and 1mg/ml purified fractions) and 210 μl of the diluted ABTS^•+^ solution. The obtained results were shown as millimolar trolox equivalent antioxidant capacity (TEAC)/mg protein. For calculating the TEAC values a calibration curve was made using different amounts of trolox (50 to 1000 μM). Absorbance was noted at 1, 2, 5, and 10 min during the reaction. The percentage reduction of the radical cation ABTS^•+^ was calculated using the following formula:

$$\%\;Reduction\;=\;\frac{Absorbance\;of\;blank-Absorbance\;of\;sample}{Absorbance\;of\;blank}\times 100$$

The TEAC values were estimated by dividing the slope value of trolox standard curve from the individual percentage reduction values of samples [35,36].

The superoxide radical (O_2_^•-^) scavenging activity of the FPLC fractions of the papain hydrolysates was analyzed by the pyrogallol autoxidation method of Marklund and Marklund [10,37] and was adapted for use in the 96-well microplates. The initial reaction mixture was prepared by adding 100 μl of the sample (in 50mM phosphate buffer, pH 6.9) to 180 μl of Tris-HCl buffer (50 mM; pH 8.2). The mixture was kept at 25 °C (10 min). Superoxide radicals were generated by adding 10 μl Pyrogallol (10 mM) prepared in 10 mM HCl). The absorbance of the final reaction mixture was read at 320 nm up to 4 minutes Phosphate buffer (50 mM, pH 6.9) was used as a blank. The O_2_^•-^ scavenging activity of ascorbic acid was also determined for comparison. The percentage of scavenged superoxide anion was estimated from the following formula:

$$\%\;O2•-\;scavenging\;=\;\frac{Slope\;of\;blank-Slope\;of\;sample}{Slope\;of\;blank}\times 100$$

### Determining ferrous chelating activity

The ability of *T*. *cordifolia* aqueous extract and digests to chelate the transition metal ion Fe^2+^ was calculated according to Wu et al.[38]. Samples (each 25 μL) were treated with 125 μL of 80 μM FeCl_2_ (final concentration in the assay solution, 40 μM). After 3 min, the reaction was started by adding 100 μL of 0.5 mM ferrozine (Final concentration of 0.2 mM). The mixtures were incubated for 15 minutes and absorbance measured (at 562 nm) [35]. The chelation ability was determined using the following equation:

$$\%\;Chelation\;=\;\frac{Absorbance\;of\;blank-Absorbance\;of\;sample}{Absorbance\;of\;blank}\times 100$$

### Liquid chromatography of papain digest

The purification of proteins/peptides present in the papain digests was done by utilizing the Fast Protein Liquid Chromatography [ÄKTA purifier Frac-950] using a size exclusion chromatography column (GE XK16, 16×100 mm) and superdex G-30 matrix with a separation range of MW 0.5–10 kDa. The matrix was packed into the column and equilibrated with phosphate buffer (0.05 M; pH 6.9) over 5 column volumes. After equilibration, 30 milligrams of double filtered (0.22 μm syringe filter) papain hydrolysate was loaded into the column. The different fractions were eluted out of the column at a specific flow rate (0.5 ml/min). Fractions were pooled together based on the protein and peptide maxima detected at 280 nm and 215 nm respectively and directly lyophilized and stored at -20°C.

### MALDI-TOF-MS and MS/MS

Lyophilized protein (fraction 9) was resuspended in 20 μl solution of 100 mM ammonium bicarbonate and 5% acetonitrile. The pH was adjusted to pH >7.5 by using 1M Tris-HCl (pH 8.0). 2 μl of 50 mM DTT was treated and the reaction mixture was kept at 65 °C (for 5 min). 2 μl of 100 mM iodoacetamide was included and kept for 30 minutes in dark at 30 °C. 2 μl of 100 ng/μl of stock trypsin (modified sequencing grade) was included and incubated overnight at 37 °C. The reaction was terminated by adjusting pH less than pH 6.0 by using 0.5% acetic acid. MALDI-TOF analysis was performed on the in-solution tryptic digest of the FPLC fraction.

MS/MS of proteins/peptides of major peaks obtained in MS spectra of fraction 9 were performed. MS and MS/MS spectra of proteins and peptides were obtained by the positive ion mode on the MALDI-TOF/TOF Mass Spectrometer (Applied Biosystems 4700 Proteomics Analyzer, Farmingham, USA). Close external mass calibration for MS was carried out using 4700 Cal Mix (Applied Biosystems). The baseline was corrected and referred for the raw data.

### Peptide sequencing

The MALDI-TOF-MS/MS data for each peptide was then processed by ‘GPS explorer^™^ software—DeNovo explorer’. The following modifications were considered: oxidation [M] and carbamidomethyl [C].

### Statistical analysis

Experiments were done with 2 or 3 independent replications. Antioxidant assays for all samples were carried out in triplicates. The obtained results were expressed in terms of mean ± standard deviation and subjected to analysis by analysis of variance (ANOVA) using Microsoft-Excel 2013 and IBM SPSS Statistics (Version 19 for Windows 8.1, SPSS Inc.) software. The significant differences (P<0.05) among individual means were calculated.

## Results and discussion

### SDS-PAGE analysis of *T*. *cordifolia* digests

**Fig 1:**
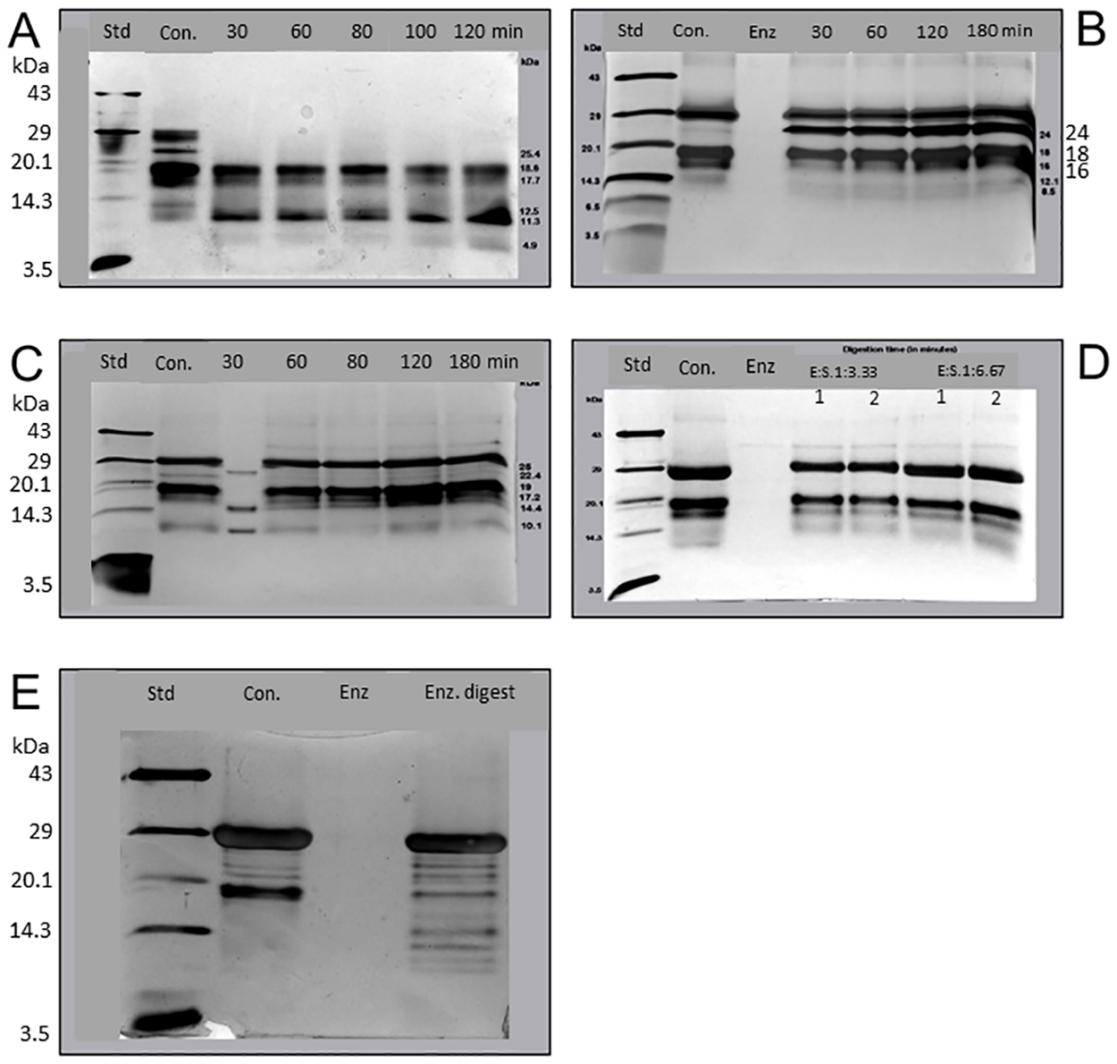
Analyzes on enzyme digests. **(A)** Tricine SDS-PAGE (14%) of *T*. *cordifolia* stem proteins treated with papain enzyme. Lane 1, marker (GeNei low molecular weight); lane 2, protein not treated with enzyme; lanes 3 to 7, protein treated with papain enzyme for different time intervals of 30 to 120 minutes; **(B)** Tricine SDS-PAGE (14%) of *T*. *cordifolia* stem proteins treated with trypsin enzyme. Lane 1, marker (GeNei low molecular weight); lane 2, protein not treated with an enzyme; lanes 3 to 7, protein treated with trypsin enzyme for time intervals of 30 to 120 minutes; **(C)** Tricine SDS-PAGE (14%) of *T*. *cordifolia* stem proteins treated with the α-chymotrypsin enzyme. Lane 1, marker (GeNei low molecular weight); lane 2, protein not treated with an enzyme; lanes 3 to 7, protein treated with the α-chymotrypsin enzyme for different time intervals of 30 to 120 minutes; **(D)** Tricine SDS-PAGE (14%) of *T*. *cordifolia* stem proteins treated with pepsin enzyme with silver staining. Lane 1, marker (GeNei low molecular weight); lane 2, protein not treated with the enzyme; lane 3, enzyme alone; lanes 4 to 7, protein treated with pepsin enzyme for different time intervals of 60 and 120 minutes, with two different enzyme:protein ratios of 1:3.33 and 1:6.67 (wt/wt); **(E)** Tricine SDS-PAGE (14%) of *T*. *cordifolia* stem proteins treated with pepsin-pancreatin enzymes with silver staining. Lane 1, marker (GeNei low molecular weight); lane 2, protein not treated with an enzyme; lane 3, enzyme alone; lane 4, protein treated with pepsin-pancreatin enzyme for 2 and 4 hours.

The extracted *T*. *cordifolia* stem proteins were digested using both plant and animal-derived digestive enzymes. Different protein profiles in the SDS-PAGE gels demonstrated the differential cleavage sites of the digestive enzymes on the *T*. *cordifolia* proteins. The results of SDS-PAGE indicate hydrolysis of 27 kDa protein band and accumulation of 11.3 kDa band along with the appearance of 4.9 kDa band demonstrating partial digestion of *T*. *cordifolia* stem proteins by papain enzyme (Fig 1(A)), as observed in the weak proteolytic effect of papain on commercial whey proteins [39]. The proteolytic effect of papain on the *T*. *cordifolia* proteins was more distinct than that of the other proteolytic enzymes used at higher than physiological ratios. While *in-vitro* simulation of proteins with trypsin enzyme (E:S = 1:5) showed the appearance of a new band at 23.8kDa molecular range in SDS-PAGE (Fig 1(B)), no profile change was observed for simulated digestion using α-chymotrypsin (E:S = 1:5, 8U/mg protein) as indicated in Fig 1(C). These results are in contrast withμ- and m-calpain digestion with trypsin and chymotrypsin indicating the enzyme inhibitory effect of *T*. *cordifolia* stem proteins [29].

The SDS-PAGE analysis of *T*. *cordifolia* proteins treated with pepsin (at E/S = 1: 3.33 and 1:6.67) indicated limited hydrolysis (Fig 1(D)) even though the enzyme/substrate ratio was higher than that resembling the physiological ratio (1:20, wt/wt, 172 U/mg). This indicates that the protein resistance to pepsin digestion is comparable with flaxseed and egg white ovalbumin digestion mimics the physiological conditions [40]. The SDS-PAGE profile of *in vitro* pepsin-pancreatin digests showed a decrease in intensity of 19kDa molecular weight proteins and appearance of new bands that indicate limited hydrolysis of these proteins as shown in Fig 1(E). Thus, the papain digests were applied for further purification and antioxidant activity analysis owing to their unique cleavage of the 27 kDa proteins among all the proteases used for *in-vitro* digestive simulation. This could be due to the combination of both endo- and exoprotease activity shown by the cysteine protease papain.

### Effect of *T*. *cordifolia* proteins on intestinal enzyme activity

**Fig 2:**
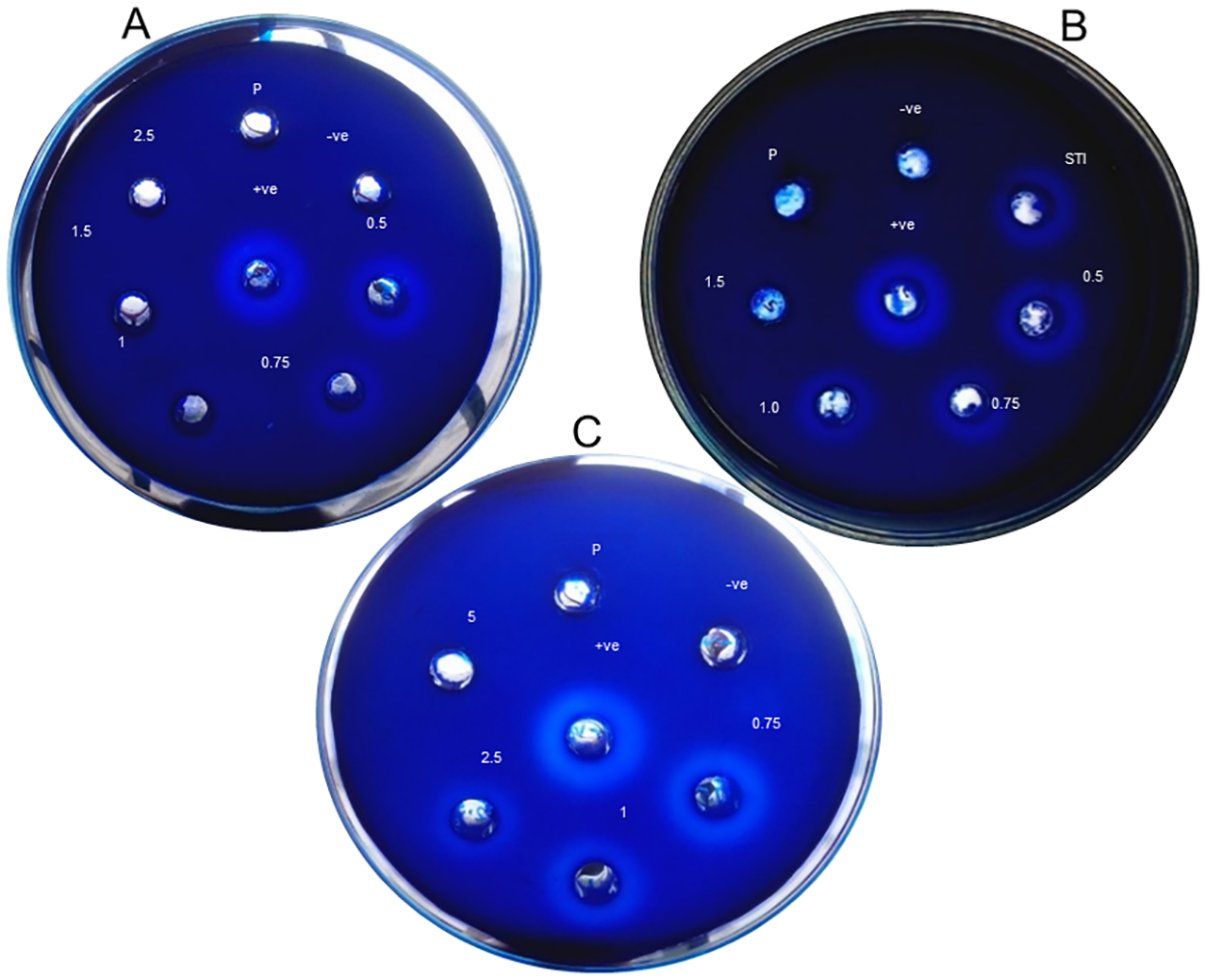
Disc diffusion plate assay showing *T*. *cordifolia* stem proteins’ effect on trypsin and α-chymotrypsin activity. The wells contained 5 ug of enzyme with varying amounts of proteins. A: Stem proteins incubated with trypsin; B: Both stem proteins and STI incubated with trypsin. C: Stem proteins incubated with α-chymotrypsin. +ive, 5 μg enzyme; -ive, 50 mM Tris-HCl buffer (pH 8.0); P, Protein sample (5 μg); 5, 5 μg enzyme + 5 μg protein (E:S = 1:1); 2.5, 5 μg enzyme + 2.5 μg protein (E:S = 2:1); 1.5, 5 μg enzyme + 1.5 μg protein (E:S = 3.33:1); 1, 5 μg enzyme + 1 μg protein (E:S = 5:1); 0.75, 5 μg enzyme + 0.75 μg protein (E:S = 6.67:1); 0.5, 5 μg enzyme + 0.5 μg protein (E:S = 10:1); STI, 5 μg enzyme + 1.5 μg Soybean Trypsin Inhibitor.

The zones of enzyme activity are formed on the gelatin plates due to the proteolytic activity of proteases on the gelatin protein. The decrease in zone diameter after adding protein indicates inhibition of enzyme activity. Gelatin disc diffusion plate assay was performed for the stem proteins which were incubated with trypsin and α-chymotrypsin enzymes in suitable buffers. The respective enzymes alone were used as positive control while the zone of activity was compared for analysis of the enzyme inhibitory activity of the proteins. Results indicate that with an increase in protein concentration with respect to the fixed amount of enzyme (5 μg), there was a reduction in the size of the zone of clearance for both trypsin and α-chymotrypsin enzymes. Complete reduction of the zone was observed at E/S ratio of 3.33:1 (wt/wt) for trypsin in comparison to that of the standard soybean trypsin inhibitor (Fig 2A and 2B) while the complete zone reduction was observed for the α-chymotrypsin incubated plates at enzyme/substrate ratio of 1:1 (wt/wt) as indicated in Fig 2C.

**Fig 3:**
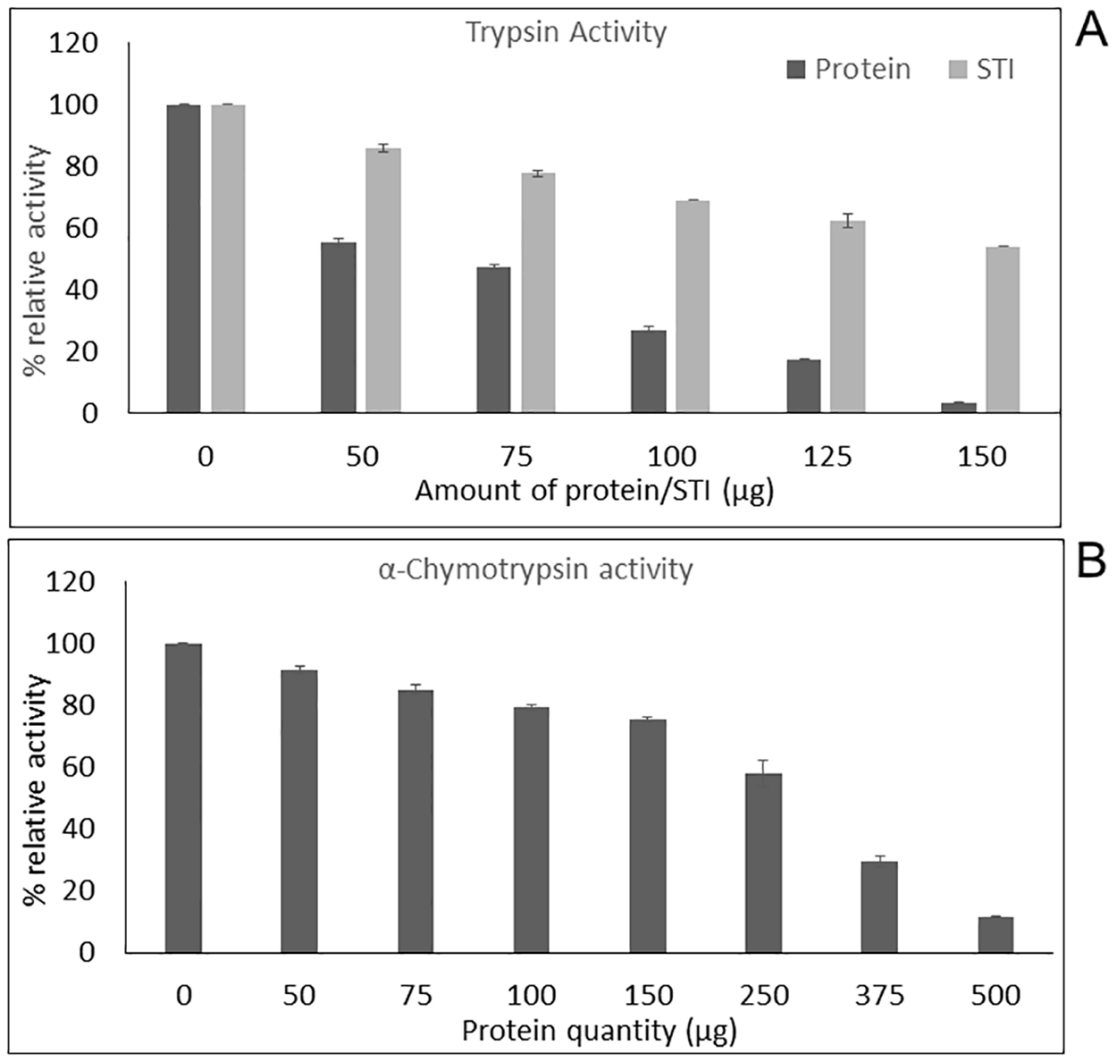
Relative activity of trypsin and α-chymotrypsin after a pre-incubation of 90 minutes with varying amounts of *T*. *cordifolia* proteins. **(A)** Increase in Trypsin enzyme inhibition with increase in amount of protein; **(B)** Increase in α-Chymotrypsin enzyme inhibition with increase in amount of protein.

Results of the spectrophotometric assays further confirmed both trypsin and α-chymotrypsin enzyme inhibitory activities of the stem proteins. 50% inhibition of trypsin was observed at a protein concentration of 72.71 micrograms (i.e. at the enzyme/protein (wt/wt) ratio of 6.9:1) higher than the standard soybean trypsin inhibitor (STI) (S2 Table, Fig 3A). 50% inhibition of α-chymotrypsin was observed at a protein concentration of 275.17 micrograms (i.e. at the enzyme/protein (wt/wt) ratio of 1.82:1) as indicated in S2 Table and Fig 3B.

### DPPH radical scavenging activity of *T*. *cordifolia* proteins and protein digests

**Fig 4:**
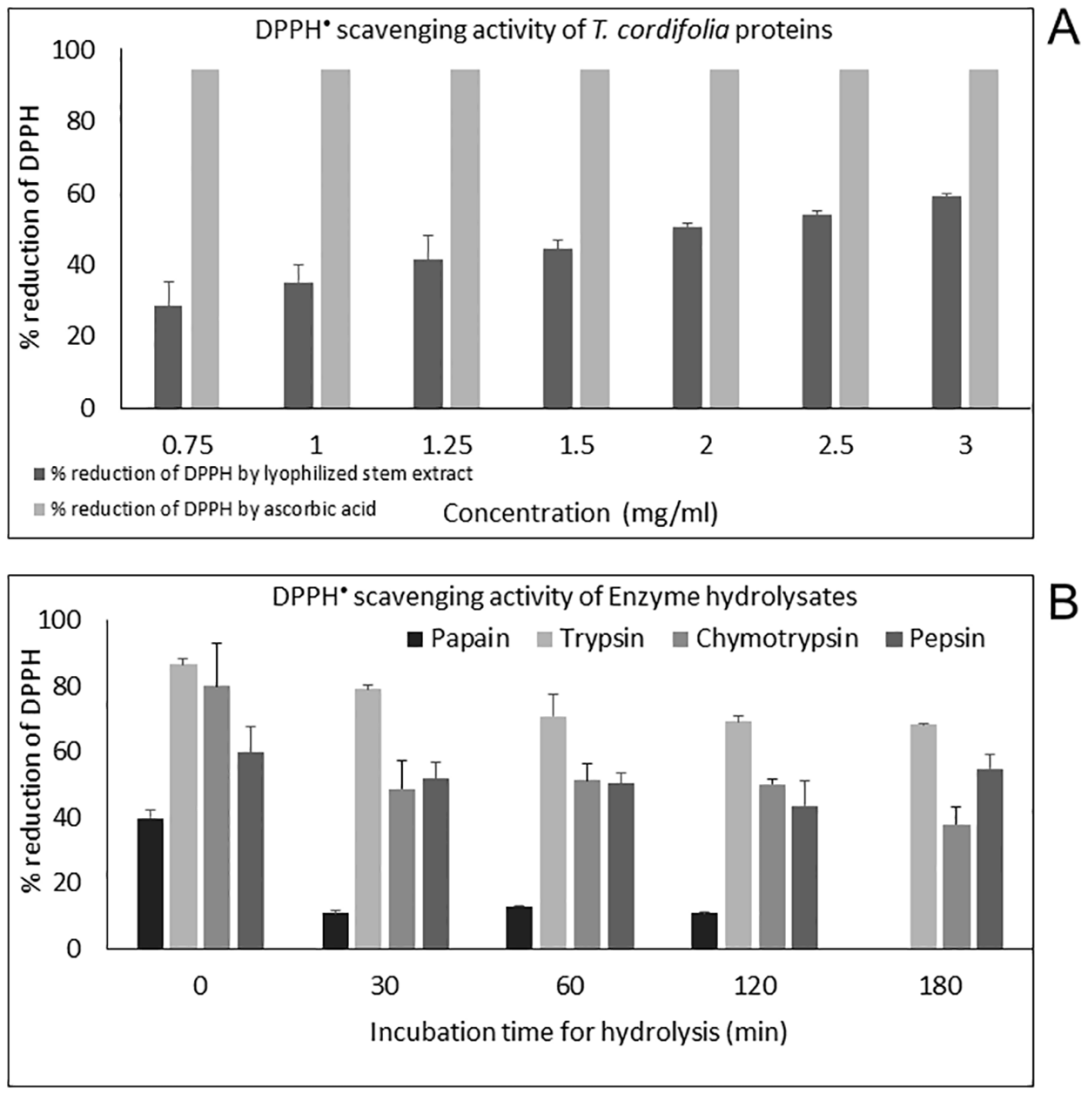
Comparison of percentage reduction of DPPH. **(A)** by different concentrations of *T*. *cordifolia* stem proteins and same concentrations of Ascorbic acid; **(B)** by *T*. *cordifolia* stem proteins hydrolyzed *in vitro* for various time intervals by different gastrointestinal enzymes (Papain, Trypsin, α-Chymotrypsin, and Pepsin).

This method involves measuring antioxidant potential by calculating the free-radical scavenging ability of antioxidants using stable free radical like 1,1-diphenyl-2-picrylhydrazyl (DPPH). Overall, increase in percentage reduction of DPPH radical with increasing concentration of *T*. *cordifolia* stem proteins (0.75 to 3 mg/ml) was observed (p = 0.000687 < 0.05) with the highest scavenging activity at the highest amount tested (3 mg/ml) (Fig 4A). The same result was shown for wheat germ protein and alfalfa leaf protein hydrolysates [41,42]. For papain hydrolysates, a decrease in DPPH radical scavenging activity compared to unhydrolyzed protein was observed but there was no change in the scavenging activity with the changes in hydrolysis time. There was a slight decrease in DPPH radical scavenging activity of proteins after hydrolysis with trypsin enzyme (79%-68%) thus, maintaining the scavenging activity even after digestion. A slight decrease was also observed in DPPH radical scavenging activity using proteins with enhancing time of pepsin hydrolysis (51%-43%) **(**S3 Table, Fig 4B). Of all the hydrolysates, trypsin hydrolysate was noticed to show the enhanced DPPH radical scavenging activity at 30 min of hydrolysis time (79.04%); the value is comparable with previous studies on alfalfa protein hydrolysates generated by commercial proteases [42].

### ABTS radical scavenging activity of *T*. *cordifolia* proteins and its enzyme hydrolysates

**Fig 5:**
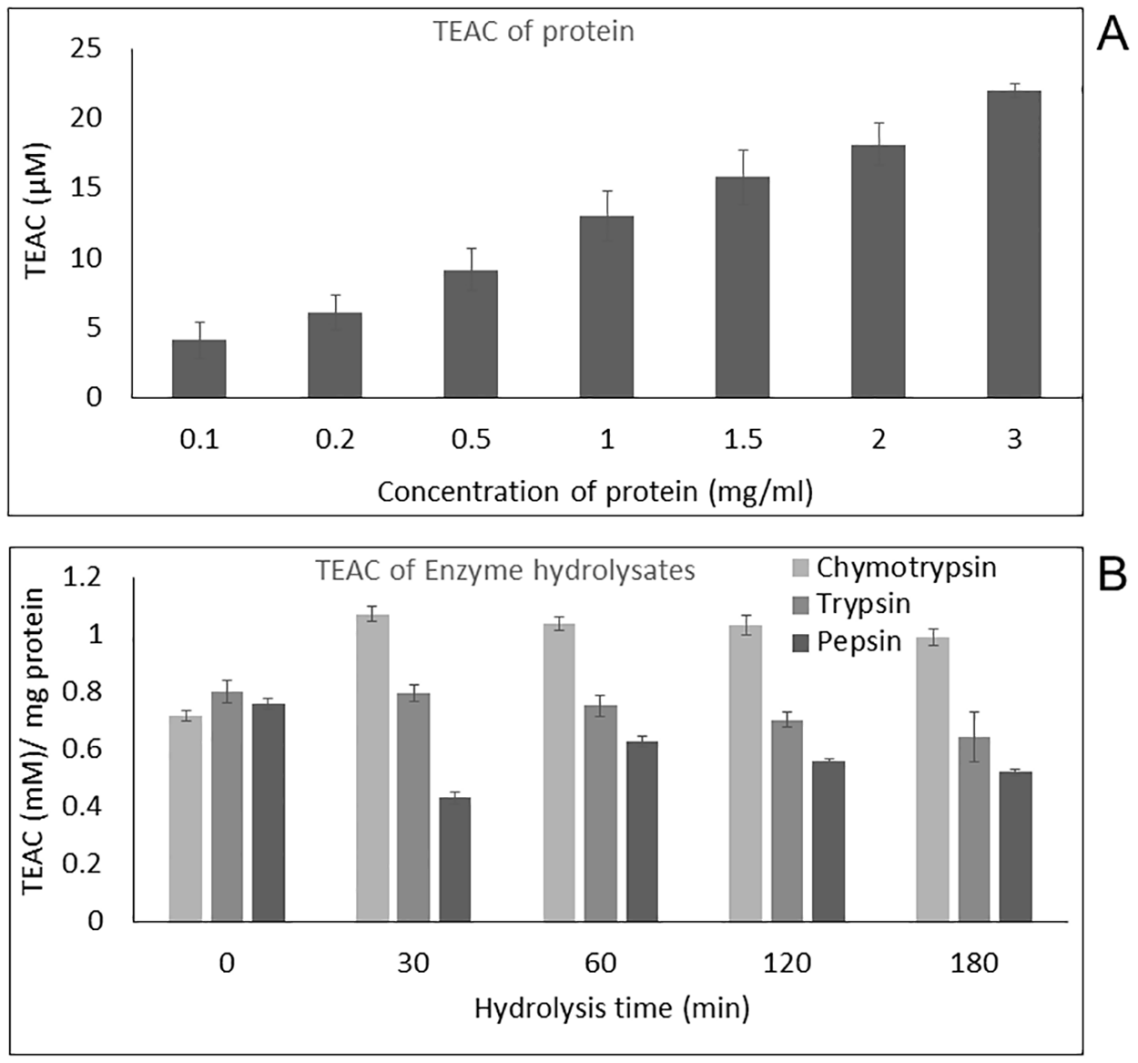
Trolox equivalent antioxidant capacity. **(A)** TEAC of *T*. *cordifolia* stem proteins using ABTS as oxidant; **(B)** TEAC of different enzyme hydrolysates (trypsin, chymotrypsin, and pepsin) of *T*. *cordifolia* stem proteins using ABTS as the oxidant.

The ABTS decolorization assay is suitable for determining the antioxidant capacities of both compounds (lipophilic and hydrophilic) due to its solubility in both aqueous and organic solvents. It has been used predominantly for assessing antioxidant activity of food antioxidants and plant and animal protein hydrolysates [35,43–47]. All enzyme digests of proteins are demonstrated to have antioxidant activity. The concentration of the digests was kept constant throughout the assay (2 μg/μl, 10 μl). The absorbance of the reaction mixtures (both test samples and control) for 10 minutes was monitored to determine the decoloration rate to attain a plateau after a reaction period (result not shown). Before hydrolysis, the *T*. *cordifolia* stem proteins showed increasing radical scavenging activity with an increase in the TEAC values (from 4.17 to 22 μM) with increasing assay concentrations of proteins (from 0.1 to 3mg/ml) **(**Fig 5A). After hydrolysis, the values were increased notably for α-chymotrypsin hydrolysates at 30 minutes of hydrolysis followed by a slight decrease with subsequent digestion times. As indicated from the p-value, trypsin hydrolysates showed a mild but significant reduction in ABTS^•+^ scavenging activity with increasing hydrolysis times (p = 0.027 < 0.05) (S4 Table, Fig 5B). For the pepsin hydrolysates, there was a drastic reduction in TEAC value at 30 min digestion (0.43 ± 0.02 mM TEAC/mg protein) with subsequent recovery of ABTS free radical scavenging activity with increasing digestion time. The α-chymotrypsin hydrolysates were the most efficient in scavenging the ABTS^•+^. There was no linear relation between hydrolysis time and radical scavenging activity for pepsin (p = 0.49 >0.05) and α-chymotrypsin digests (p = 0.45 >0.05). Papain digest was not analyzed for ABTS scavenging activity because it formed a turbid solution with the ABTS reaction mixture.

### Ferrous ion chelating activity of *T*. *cordifolia* proteins and protein hydrolysates

**Fig 6:**
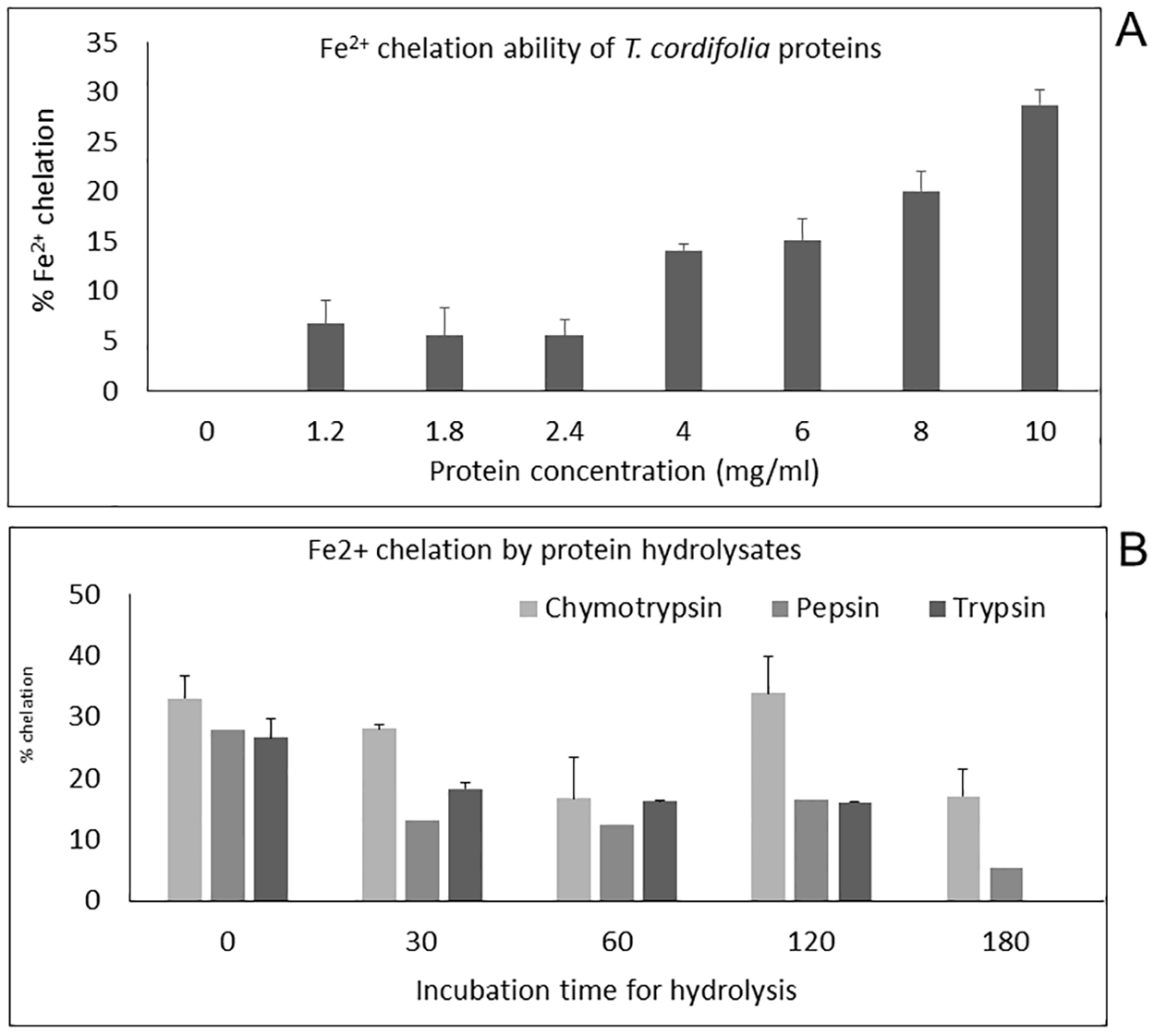
Fe^2+^ chelating activity. **(A)** *T*. *cordifolia* stem proteins; **(B)** enzyme hydrolysates (trypsin, chymotrypsin, and pepsin) of *T*. *cordifolia* stem proteins at a concentration of 4 mg/ml for all hydrolysis time.

The catalyzing activity of transition metal ions like cuprous ions (Cu^2+^) and ferrous ions (Fe^2+^) in the generation of reactive oxygen species in vivo has been well documented [48]. Fe^2+^ ions can catalyze the Haber-Weiss reaction to induce superoxide formation which results in the formation of hydroxyl radical. Hydroxyl radicals can oxidize many vital biomolecules that lead to lipid peroxidation and DNA damage. Fe^2+^ ion is considered the primary molecule involved in the Fenton reaction which also produces the highly oxidizing hydroxyl radicals [49]. Therefore, it can be inferred that the chelation of transition metal ions by antioxidant proteins or peptides would reduce the oxidation reaction and could go a long way in ameliorating the oxidative damage. The chelating activity of the *T*. *cordifolia* stem proteins is demonstrated in Fig 6A. An increase in the Fe^2+^ chelating ability of the proteins (7–29%) was noticed with increasing concentrations (0.6 mg/ml– 10 mg/ml) as indicated by the p-value (p = 0.000024 < 0.05). The ferrous ion chelation by the protein digests was found to be related to the enzyme used for digestion. Of all the enzyme digests, α-chymotrypsin had the highest chelating activity (4 mg/ml α-chymotrypsin digests showed 33.92% ferrous ion chelation at 120 min hydrolysis). The pepsin digestion resulted with a decrease in the Fe^2+^ ion chelating activity when compared with the reaction control, this kind of activity having been reported for buckwheat protein in vitro digests [35]. For trypsin, there was a slight and continuous decrease in activity with increasing digestion time (p = 0.026 < 0.05) in comparison to the control reaction. There was no linear correlation between the time of hydrolysis and the metal ion chelating activity of chymotrypsin digest as indicated by the p-value (p = 0.79 > 0.05) (Fig 6B). The changes in the rate of ferrous ion chelation could probably because of the disruption of the iron-binding structure of the proteins due to hydrolysis or the trypsin and α-chymotrypsin inhibitory activities of the *T*. *cordifolia* proteins resulting in a decrease in a number of iron-binding sites available in the protein structure [35]. The availability of the metal ion binding functional amino acid residues like N-terminal histidine at different times of hydrolysis plays a major role in metal ion chelation [50]. The increase in ferrous ion chelating activity of the heated reaction controls of both hydrolysates) might be because of the denaturation of protein after boiling resulted in the formation of active protein fractions capable of binding metal ions. Consequently, the metal chelating ability of the protein digests may influence other mechanisms of antioxidant activity [42,51,38]. The metal chelating activity of *T*. *cordifolia* protein digests was compared with the strong chelator EDTA, whose chelating capacity was 89.94% at 0.16 mg/mL. Papain hydrolyzate formed a turbid mixture with the ferrous ion chelation reaction mixture and hence could not be tested for antioxidant activity.

### Purification of proteins/peptides

The fractions obtained by FPLC (size exclusion chromatography) of papain digested *T*. *cordifolia* stem proteins were pooled into 18 fractions (S5 Table based on the protein and peptide peaks obtained in the chromatogram **(**S1 Fig) and were lyophilized.

### Radical scavenging activity of FPLC fractions of papain digests

**Fig 7:**
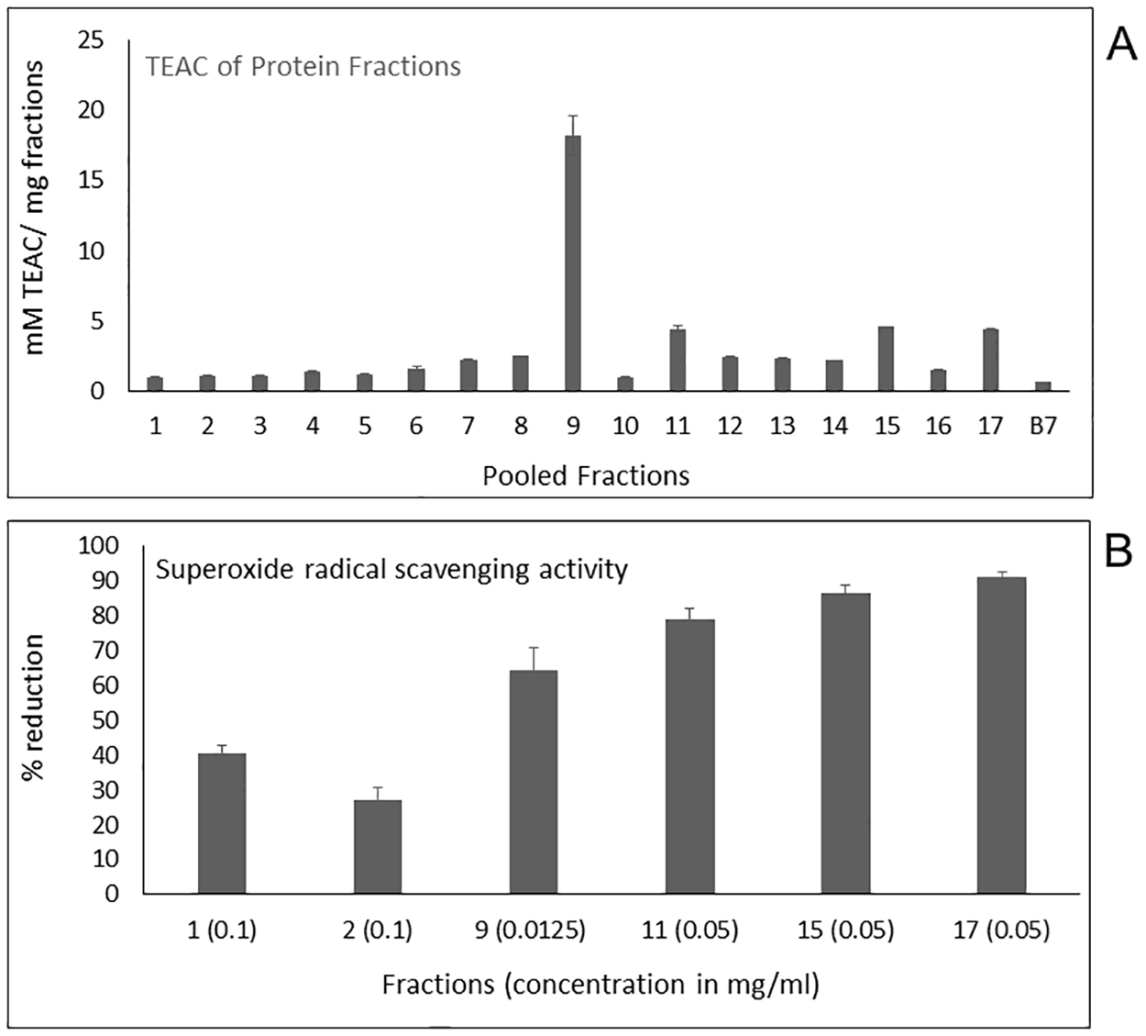
Radical scavenging activity of fractions obtained by FPLC of papain hydrolysate. **(A)** TEAC of fractions; **(B)** O^2-^ radical scavenging activity of fractions. The X-axis represents fraction numbers with its concentration (mg/ml) used for the assay in parenthesis.

The strong scavenging of the relatively stable free radical, ABTS is a strong indicator of the high antioxidant effectiveness of a molecule or compound. All FPLC fractions of the papain digests exhibited ABTS free radical scavenging activity except fraction 18. Among all the fractions, fraction 9 (18.20 ± 1.37 mM TEAC/mg protein) showed the highest scavenging activity, while the TEAC values were similar for fractions 11 (4.43 ± 0.21 mM TEAC/mg protein), 15 (4.57 ± 0.02 mM TEAC/ mg protein), and 17 (4.38 ± 0.07 mM TEAC/mg protein) indicating the occurrence of similar ABTS^•+^quenching activity (S5 Table, Fig 7A). The ABTS radical scavenging of the mentioned fractions (9, 11, 15, 17) is considerably higher than that of zein hydrolysate (2.5 mM TEAC/mg protein) [52] and buckwheat protein in vitro digests (2 mM TEAC/mg protein) [35].

The toxic superoxide radical is formed in many autoxidation reactions in vivo and the electron transport chain. It participates in numerous biological reactions generating many reactive oxygen species(ROS) like hydrogen peroxide, peroxynitrite (ONOO^–^), hypochlorous acid (HOCl), and the most reactive ROS *in vivo*, the hydroxyl radical (OH^•^) via the Haber-Weiss reaction [5]. All generated free radicals are major perpetrators of oxidative damage [53]. Studies on the scavenging of these free radicals are important in understanding ROS defense mechanisms and developing more effective antioxidant drugs. Fractions 9, 11, 15, and 17 showed a strong superoxide radical scavenging activity, while fractions 1 and 2 demonstrated a moderate activity (S5 Table, Fig 7B). Fraction 9 has the highest superoxide radical scavenging activity completely scavenging the free radical at both 0.1 mg/ml and 0.05 mg/ml. Fraction 9 at 0.0125 mg/ml showed 64.15 ± 6.51% superoxide scavenging activity, making it a more effective radical scavenger when compared with the reported 69.15% at 2 mg/ml superoxide scavenging activity of purified chickpea hydrolysate fractions [54], and 45% at 1 mg/ml scavenging activity of mushroom-derived antioxidant peptides [55]. Fractions 15 (86.46 ± 2.51%) and 17 (91.01 ± 1.69%) had similar free radical quenching activity followed by fraction 11 (79.09 ± 2.95%), all were at 0.05 mg/ml. The superoxide radical scavenging activity of the fractions 9, 11, 15, and 17 are higher than that of alfalfa leaf peptides (67% at 0.9 mg/ml); purified whey protein fraction (50% at 9.3 mg/ml); and pepsin hydrolysate of barley hordein (20% at 0.5 mg/ml) [42,56,57].

Similarity in both ABTS and O_2_^•-^ scavenging activities of fractions 11, 15, 17 indicate the effect of similar antioxidant peptides released by papain enzyme hydrolysis. The peptides may or may not have similar sizes due to different elutions in the FPLC (size exclusion) column but may have common amino acid composition indicated by the same scavenging activity. Results obtained displayed that fraction 9 has strong free radical scavenging activities and has the advantage to be used as a natural antioxidant source.

The SDS-PAGE analysis of the active fractions 9, 11, 15, and 17 was done to check the profile but the proteins/peptides were not detected in 14% SDS-PAGE because of the low molecular weight of these proteins/peptides (S2 Fig).

### Amino acid of radical scavenging peptides from active fraction 9

Numerous peptides were expected to be produced on papain hydrolysis of *T*. *cordifolia* proteins since the papain enzyme has both endo- and exopeptidase activities. Matrix-assisted laser desorption/ionization time-of-flight mass spectrometry (MALDI-TOF-MS) was used to recognize the peptides present in the FPLC purified fractions of the papain digest (S3 Fig).

Although to a lesser extent, biologically active food-derived peptides are widely described by means of MALDI-MS. Antioxidant peptides in the mass range of m/z 4000–7000 have been detected in the WSE of Mungoong, a traditional Thai fishery product produced by the cephalothorax of shrimp [58]. Lunasin, a promising chemo-preventive peptide from soybean was characterized by MALDI-MS [59].

Fraction 9 showing highest radical scavenging activity was attempted by MS/MS and de-novo sequencing and the most abundant peptide having highest peak in MALDI-MS spectra was found to be of 1678.76 Da molecular weight with a possible sequence of ‘VLYSTPVKMWEPGR’ having a De novo/MS BLAST score of 78.79. Two other peptides present in high concentrations were identified to have a molecular mass of 1450.71 Da and 1023.51 Da, with possible sequences of ‘VITVVATAGSETMR’ and ‘HIGININSR’ having De novo/MS BLAST scores of 78.52 and 81.18 respectively (S4 Fig, S6 Table).

Antioxidant activity of amino acids has been attributed to their structure, composition, and hydrophobicity [50,60]. Several studies have shown a high relation with certain amino acid residues and the antioxidant activity of peptides. Hydrophobic amino acids like Ala, Pro, and Phe might cause for having high free radical scavenging activity with peptides. Hydrophobic amino acids like Leu or Val, if located at the N-terminus of a peptide, are important contributors to its antioxidant activity, with the assumption that Leu has long aliphatic side-chain group favoring interactions with acyl chains of susceptible fatty acids [50,61,62]. Amino acids having aromatic residues (Trp, Tyr) can act as direct radical scavengers of electron-deficient radicals with their phenolic and indolic groups donating protons to them [63,64]. Peptides containing His and Lys have also been noted to be a high antioxidant activity [50,65]. Histidine-containing peptides have antioxidant activity through different mechanisms like hydroxyl radical scavenging, transition-metal ion chelation, and quenching of singlet oxygen and other active oxygen species via decomposition of the imidazole ring with Histidine [50,66,67]. The radical scavenging hexapeptide with the sequence Tyr-Phe-Tyr-Pro-Glu-Leu was isolated from casein hydrolysate by Suetsuna et al. [68]. The high activity was attributed to the Glu-Leu sequence (C-terminal amino acid) in the peptide. A peptide (His-Gly-Pro-Leu-Gly-Pro-Leu) was isolated from fish-skin gelatin by Mendis et al. [69] and its high antioxidant activity attributed the presence of Leu, and His and the repeating amino acid residues of Gly-Pro are the possible reason for its high activity. In addition, a low molecular weight fraction with strong superoxide radical scavenging activity was isolated from chickpea protein hydrolysate by Li et al. [54]. It was noticed that the fraction had the significantly enhanced concentrations of Phe, Ile, Leu, and Val when compared to other fractions and so it explained that the superoxide scavenging activity might be related to the amino acids with hydrophobicity.

Fraction 9 has a very high superoxide and ABTS radical scavenging activity. This may be correlated to the increased levels of total hydrophobicity of amino acids in the peptides of active fraction 9. Two prominent peptides in fraction 9 (VLYSTPVKMWEPGR and VITVVATAGSETMR) contained Val at their N-terminus, in which VLYSTPVKMWEPGR comprising the antioxidant amino acids Pro, Gly, Leu, and Trp. The purified peptide with sequence HIGININSR contained three residues of the hydrophobic amino acid Isoleucine. Although a definite relationship between the structure and activity cannot be established for the *T*. *cordifolia* peptides, it can be explained that the amino acids in the peptide sequences are having low molecular weight, especially in fractions 9, 11, 15, and 17 that had a strong radical scavenging activity with the hydrophobic amino acids responsible for both superoxide and ABTS radical scavenging activity.

## Conclusion

*T*. *cordifolia* stem proteins showed a strong trypsin inhibitory activity (greater than standard soybean trypsin inhibitor), while it also displayed α-chymotrypsin inhibition. Both the protein extracts and protein hydrolysates showed considerable DPPH and ABTS radical scavenging activities, and moderate ferrous ion chelating activity. The strong gastrointestinal enzyme inhibition coupled with a high antioxidant activity suggests a probable prolonged antioxidant effect of the stem proteins after ingestion. The extremely high ABTS and superoxide scavenging activities of papain digest fractions indicated that the lower molecular weight peptides were efficient free radical scavengers than the higher proteins and peptides, owing to the hydrophobic and aromatic amino acids composition. Since *T*. *cordifolia* is already an important composition of many traditional Indian medicine formulations, both its purified stem proteins and the derived peptides by enzyme hydrolysis could be incorporated into food products or nutraceuticals or developed to be a safe and efficient drug for treating oxidative stress and related disorders.

## Supporting information

S1 Fig FPLC profile of papain digest of *T*. *cordifolia* stem proteins. Superdex 30 showing, peptides (at 215 nm) and proteins (at 280 nm).
(DOCX)

S2 Fig Tricine SDS-PAGE (14%) of FPLC fractions of *T*. *cordifolia* stem proteins treated with papain enzyme (2 hr). Lane 1, marker (GeNei low molecular weight); lane 2, a protein not treated with an enzyme; lane 3, a protein digested with papain enzyme for 2 hours; lane 4, fraction 9; lane 5, fraction 11; lane 6, fraction 15; lane 7, fraction 17.
(DOCX)

S3 Fig MALDI MS profile of fraction 9. From the size exclusion chromatography of papain digest of *T*. *cordifolia* stem proteins.
(DOCX)

S4 Fig MS/MS spectra and De novo/MS BLAST search. Three peptides identified by MALDI-MS of fraction 9.
(DOCX)

S1 Table Conditions maintained during enzymatic hydrolysis of *T*. *cordifolia* stem proteins with various enzymes. (DOCX)

S2 Table Comparison of relative activity of trypsin and α-chymotrypsin enzymes after adding different amounts of protein and soybean trypsin inhibitor (STI). (DOCX)

S3 Table Percent reduction of DPPH by papain, trypsin, α-chymotrypsin and pepsin hydrolysates of *T*. *cordifolia* stems proteins. (DOCX)

S4 Table TEAC (using ABTS as oxidant) and Fe^2+^ chelation activity of enzyme digests of *T*. *cordifolia* stem proteins *SD: Standard deviation. (DOCX)

S5 Table Pooled fractions based on protein and peptide maxima (280 nm and 215 nm respectively); and their TEAC values and superoxide radical scavenging activity. (DOCX)

S6 Table Sequence of five major peptides identified in MALDI-MS spectra of fraction 9. (DOCX)
