# Supplementary material for: Separation and identification of bioactive peptides from stem of Tinospora cordifolia (Willd.) Miers
Source: PLoS One. 2018 Mar 1;13(3):e0193717. doi: 10.1371/journal.pone.0193717 (PMC5832316; doi:10.1371/journal.pone.0193717)
Supplement: S1 Fig — Superdex 30 showing, peptides (at 215 nm) and proteins (at 280 nm). (DOCX) [file pone.0193717.s001.docx]

**S1 Fig FPLC profile of papain digest of *T. cordifolia* stem proteins**. Superdex 30 showing, peptides (at 215 nm) and proteins (at 280 nm).


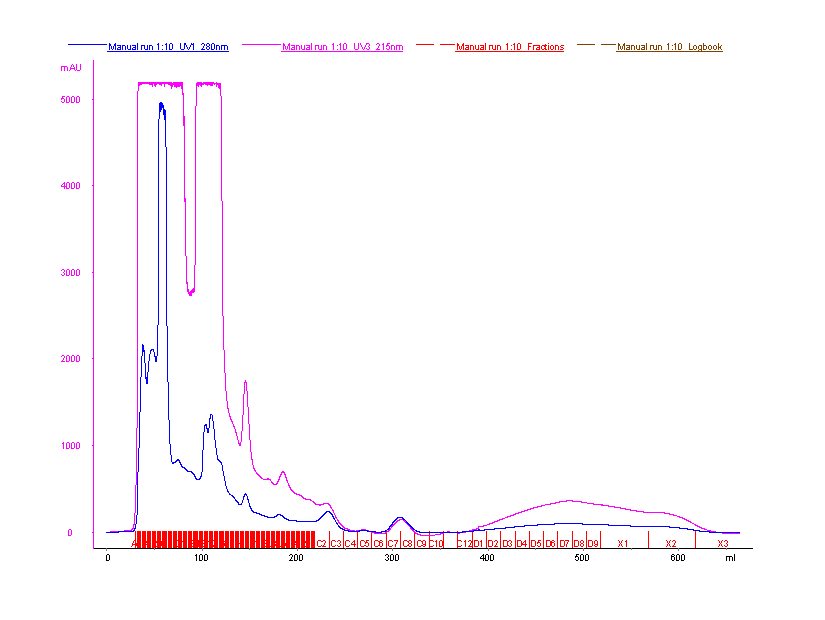


**9**

**15**

**17**

**11**
