# Supplementary material for: Separation and identification of bioactive peptides from stem of Tinospora cordifolia (Willd.) Miers
Source: PLoS One. 2018 Mar 1;13(3):e0193717. doi: 10.1371/journal.pone.0193717 (PMC5832316; doi:10.1371/journal.pone.0193717)
Supplement: S2 Fig — Lane 1, marker (GeNei low molecular weight); lane 2, a protein not treated with an enzyme; lane 3, a protein digested with papain enzyme for 2 hours; lane 4, fraction 9; lane 5, fraction 11; lane 6, fraction 15; lane 7, fraction 17. (DOCX) [file pone.0193717.s002.docx]

**
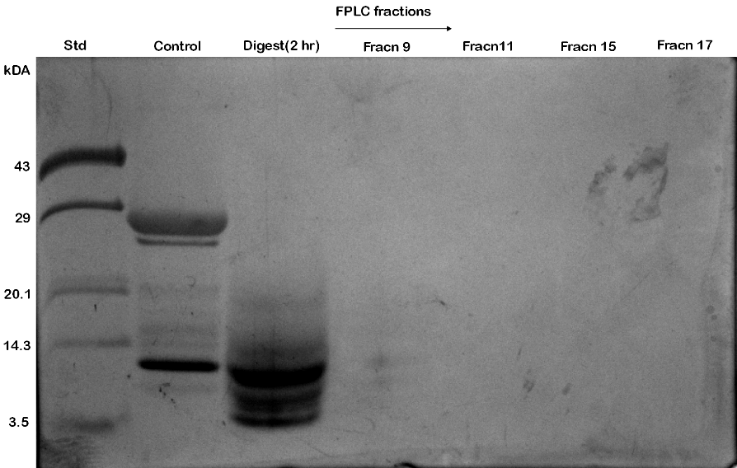
S2 Fig Tricine SDS-PAGE (14%) of FPLC fractions** of *T. cordifolia* stem proteins treated with papain enzyme (2 hr). Lane 1, marker (GeNei low molecular weight); lane 2, a protein not treated with an enzyme; lane 3, a protein digested with papain enzyme for 2 hours; lane 4, fraction 9; lane 5, fraction 11; lane 6, fraction 15; lane 7, fraction 17.
