# Supplementary material for: Separation and identification of bioactive peptides from stem of Tinospora cordifolia (Willd.) Miers
Source: PLoS One. 2018 Mar 1;13(3):e0193717. doi: 10.1371/journal.pone.0193717 (PMC5832316; doi:10.1371/journal.pone.0193717)

**S3 Fig MALDI MS profile of fraction 9**. From the size exclusion chromatography of papain digest of *T. cordifolia* stem proteins.


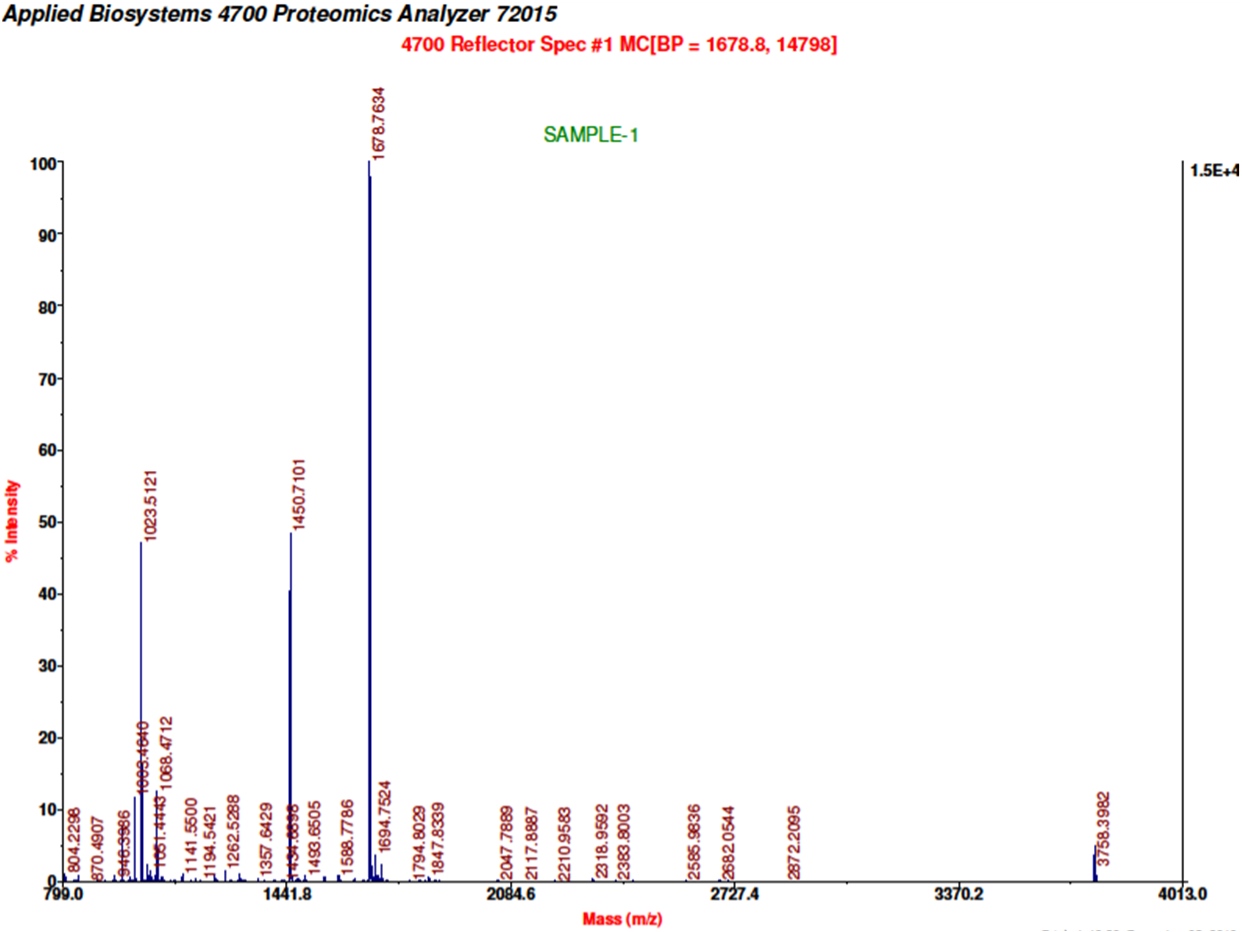

Supplement: S3 Fig — From the size exclusion chromatography of papain digest of T. cordifolia stem proteins. (DOCX) [file pone.0193717.s003.docx]
