# Supplementary figures and images for: Separation and identification of bioactive peptides from stem of Tinospora cordifolia (Willd.) Miers
Source: PLoS One. 2018 Mar 1;13(3):e0193717. doi: 10.1371/journal.pone.0193717 (PMC5832316; doi:10.1371/journal.pone.0193717)

**S4 Fig MS/MS spectra and De novo/MS BLAST search**. Three peptides identified by MALDI MS of fraction 9.


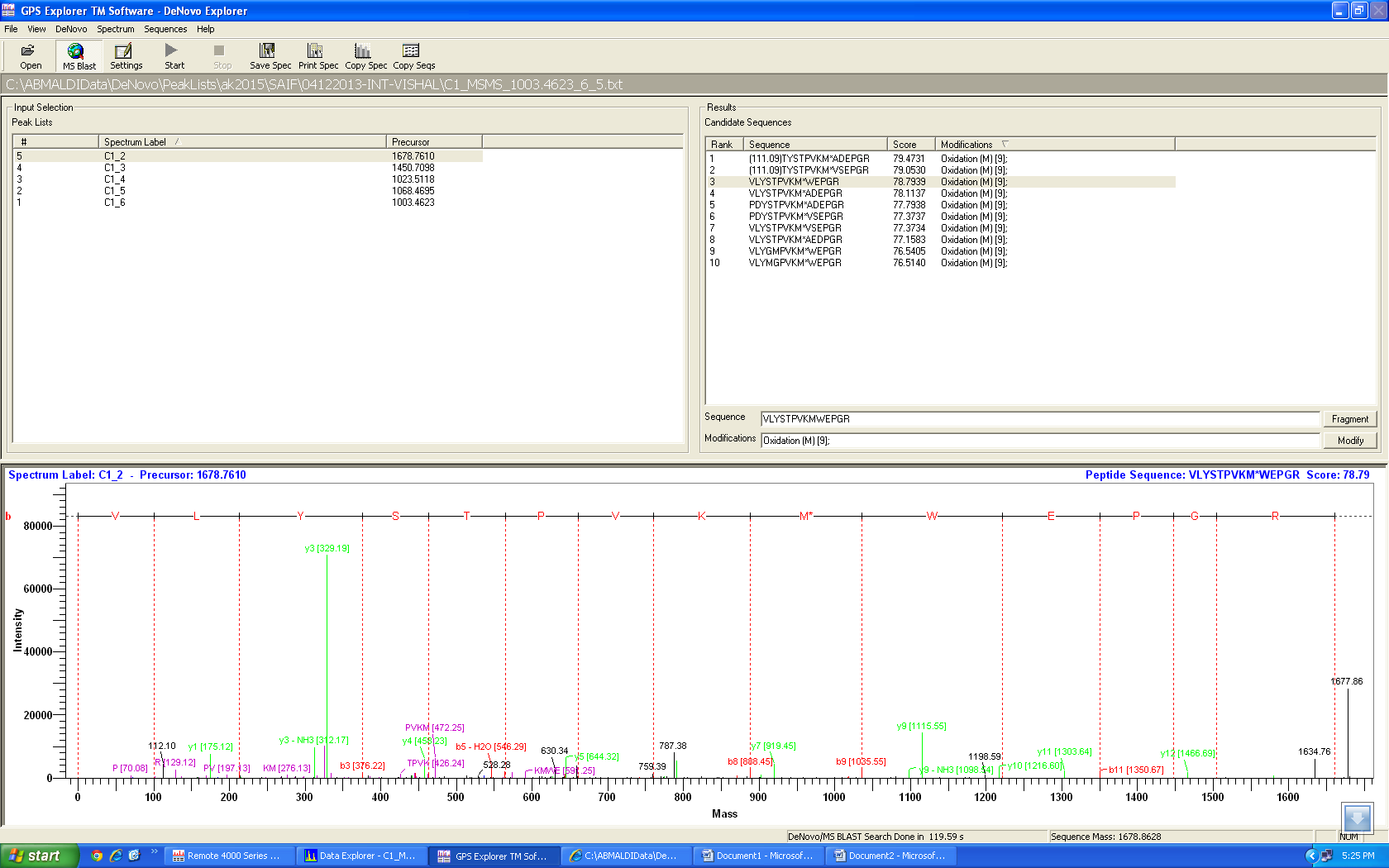

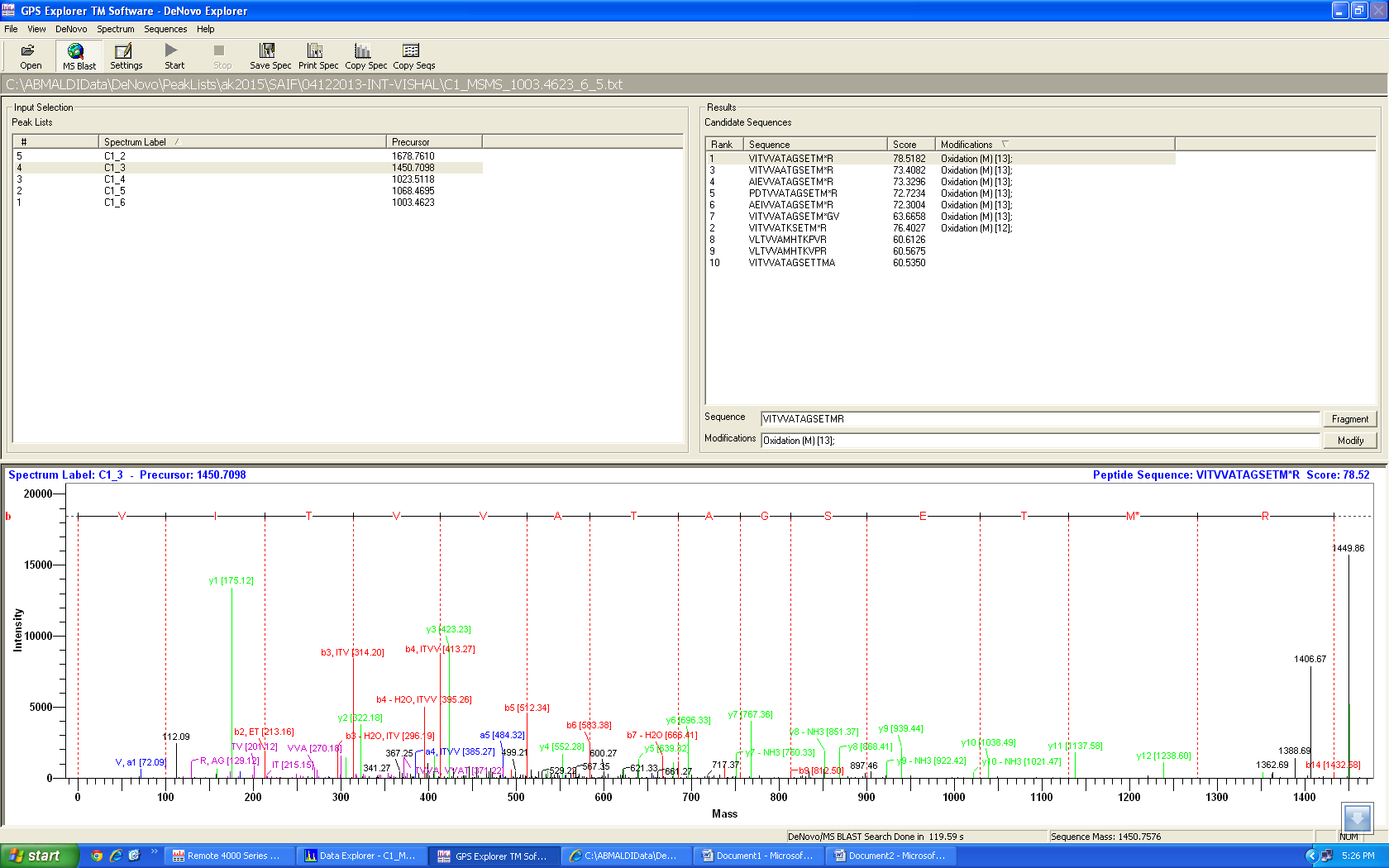

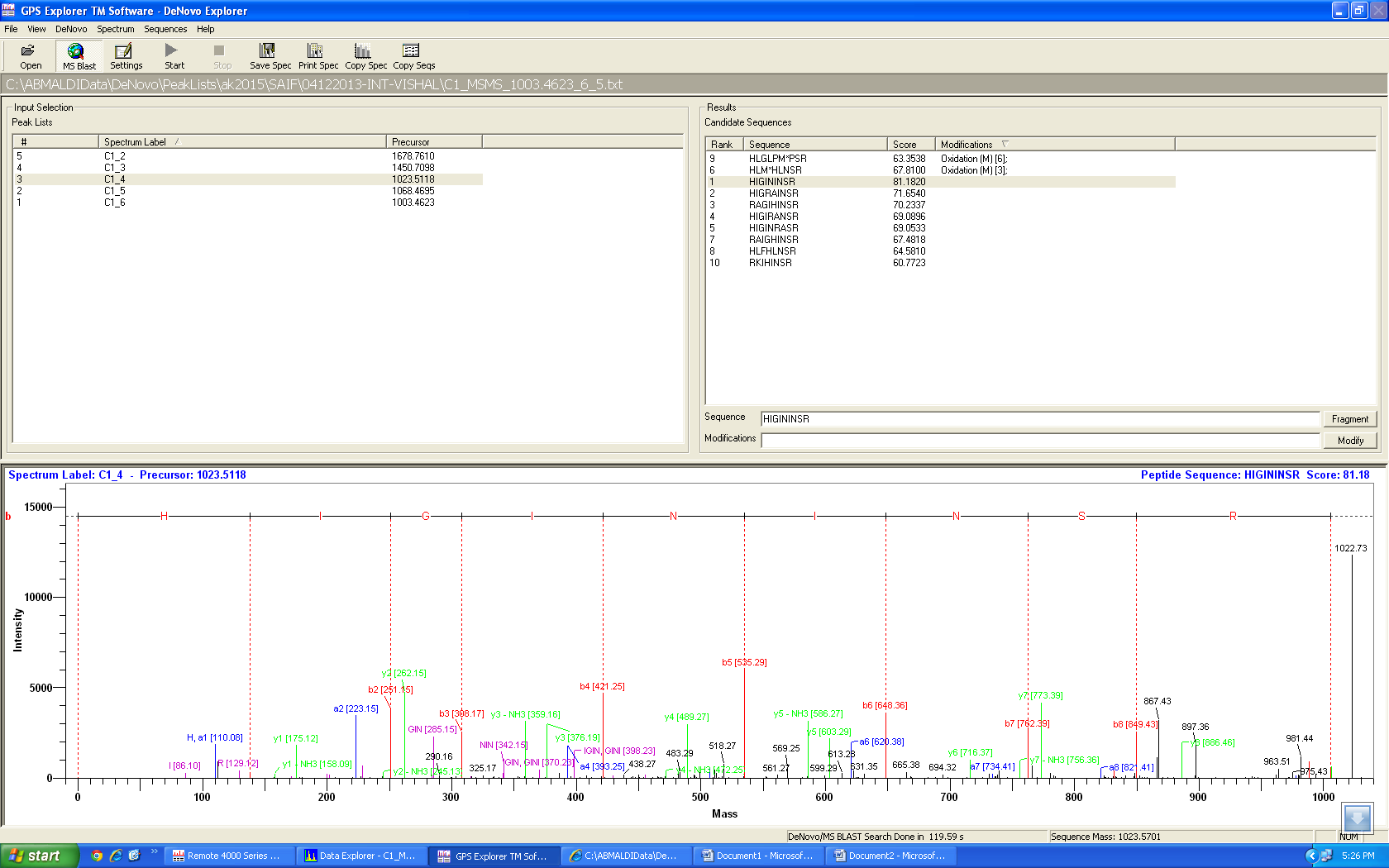

Supplement: S4 Fig — Three peptides identified by MALDI-MS of fraction 9. (DOCX) [file pone.0193717.s004.docx]
