# Supplementary material for: Separation and identification of bioactive peptides from stem of Tinospora cordifolia (Willd.) Miers
Source: PLoS One. 2018 Mar 1;13(3):e0193717. doi: 10.1371/journal.pone.0193717 (PMC5832316; doi:10.1371/journal.pone.0193717)
Supplement: S1 Table — (DOCX) [file pone.0193717.s005.docx]

**S1 Table Conditions maintained during enzymatic hydrolysis of *T. cordifolia* stem proteins with various enzymes**

| Enzyme | Buffer | pH | Enzyme:Substrate (wt/wt) | Temperature (⁰C) | Time (minutes) |
| --- | --- | --- | --- | --- | --- |
| Papain | 50 mM sodium phosphate | 6.8 | 1:100 | 37⁰C | 30 to 120 |
| Pepsin | 10mM HCl + 30 mM NaCl | 2.2 | 1:3.33 | 37⁰C | 60 & 120 |
| α chymotrypsin | 50 mM tris-HCl | 7.8 | 1:5 | 37⁰C | 30 to 180 |
| trypsin | 50 mM tris-HCl | 8.0 | 1:5 | 37⁰C | 30 to 180 |
| pepsin-pancreatin | 10 mM HCl + 30 mM NaCl | 2.2 | 1:3.33 | 37⁰C | 120 |
|  | + 0.9 M NaHCO_3_ | 7.5 | 1:5 | 37⁰C | 240 |
