# Supplementary material for: Separation and identification of bioactive peptides from stem of Tinospora cordifolia (Willd.) Miers
Source: PLoS One. 2018 Mar 1;13(3):e0193717. doi: 10.1371/journal.pone.0193717 (PMC5832316; doi:10.1371/journal.pone.0193717)
Supplement: S2 Table — (DOCX) [file pone.0193717.s006.docx]

**S2 Table** **Comparison of relative activity of Trypsin and α-Chymotrypsin enzymes after adding different amounts of protein and Soybean trypsin inhibitor (STI).**

| amount of protein/STI (ug) | relative trypsin activity with protein ± SD (n=6) | relative trypsin activity with STI ± SD (n=6) | relative α-Chymotrypsin activity with protein ± SD (n=6) |
| --- | --- | --- | --- |
| 0 | 100.00 ± 0 | 100.00 ± 0 | 100.00 ± 0 |
| 50 | 55.21 ± 1.34 | 85.77 ± 1.49 | 91.35 ± 1.2 |
| 75 | 47.37 ± 0.74 | 77.56 ± 1.09 | 85.07 ± 1.55 |
| 100 | 26.97 ± 1.13 | 68.90 ± 0.04 | 79.24 ± 1.21 |
| 125 | 17.33 ± 0.08 | 62.38 ± 2.36 | _-_ |
| 150 | 3.26 ± 0.4 | 53.85 ± 0.05 | 75.45 ± 0.8 |
| 250 | - | - | 58.08 ± 4.34 |
| 375 | - | - | 29.67 ± 1.62 |
| 500 | - | - | 11.56 ± 0.05 |
