# Supplementary material for: Separation and identification of bioactive peptides from stem of Tinospora cordifolia (Willd.) Miers
Source: PLoS One. 2018 Mar 1;13(3):e0193717. doi: 10.1371/journal.pone.0193717 (PMC5832316; doi:10.1371/journal.pone.0193717)
Supplement: S3 Table — (DOCX) [file pone.0193717.s007.docx]

**S3 Table** **Percent reduction of DPPH by papain, trypsin, α-chymotrypsin and pepsin hydrolysates of *T. cordifolia* stems proteins.**

| hydrolysis time (minutes) | DPPH^•^scavenging (%) ± standard deviation | | | |
| --- | --- | --- | --- | --- |
|  | papain hydrolysate | trypsin hydrolysate | α chymotrypsin hydrolysate | pepsin hydrolysate |
| 0 | 39.79 ± 2.53 | 86.56 ± 1.71 | 80.08 ± 13.24 | 59.85 ± 7.62 |
| 30 | 10.81 ± 0.87 | 79.04 ± 1.32 | 48.77 ± 8.67 | 51.79 ± 4.95 |
| 60 | 12.77 ± 0.37 | 70.86 ± 6.69 | 51.24 ± 5.04 | 50.35 ± 3.41 |
| 120 | 10.72 ± 0.63 | 69.22 ± 1.88 | 50.1 ± 1.37 | 43.45 ± 7.56 |
| 180 | - | 68.29 ± 0.14 | 37.94 ± 5.13 | 54.77 ± 4.21 |
