# Supplementary material for: Separation and identification of bioactive peptides from stem of Tinospora cordifolia (Willd.) Miers
Source: PLoS One. 2018 Mar 1;13(3):e0193717. doi: 10.1371/journal.pone.0193717 (PMC5832316; doi:10.1371/journal.pone.0193717)
Supplement: S4 Table — (DOCX) [file pone.0193717.s008.docx]

**S4 Table** **TEAC (using ABTS as oxidant) and Fe^2+^ chelation activity of enzyme digests of *T. cordifolia* stem proteins *SD: standard deviation.**

| time of hydrolysis (minutes) | mM TEAC / mg of protein digest | | | percentage chelation of Fe^2+^ by enzyme hydrolysates (4 mg/ml) | | |
| --- | --- | --- | --- | --- | --- | --- |
|  | trypsin ± SD* | α-chymotrypsin ± SD* | pepsin ± SD* | trypsin ± SD* | chymotrypsin ± SD* | pepsin ± SD* |
| 0 | 0.8 ± 0.04 | 0.72 ± 0.02 | 0.76 ± 0.02 | 26.704 ± 3.07 | 33.13 ± 0.71 | 27.97 |
| 30 | 0.8 ± 0.03 | 1.07 ± 0.03 | 0.43 ± 0.02 | 18.287 ± 1.12 | 28.19 ± 6.67 | 13.10 |
| 60 | 0.75 ± 0.03 | 1.04 ± 0.03 | 0.63 ± 0.02 | 16.289 ± 0.22 | 16.80 ± 6.13 | 12.38 |
| 120 | 0.7 ± 0.03 | 1.03 ± 0.03 | 0.56 ± 0 | 16.105 ± 0.08 | 33.92 ± 4.48 | 16.49 |
| 180 | 0.64 ± 0.09 | 0.99 ± 0.03 | 0.52 ± 0.01 | Negligible chelation | 17.09 ± 5.29 | 5.38 |
