# Supplementary material for: Separation and identification of bioactive peptides from stem of Tinospora cordifolia (Willd.) Miers
Source: PLoS One. 2018 Mar 1;13(3):e0193717. doi: 10.1371/journal.pone.0193717 (PMC5832316; doi:10.1371/journal.pone.0193717)
Supplement: S5 Table — (DOCX) [file pone.0193717.s009.docx]

**S5 Table Pooled fractions based on protein and peptide maxima (280 nm and 215 nm respectively); and their TEAC values and Superoxide radical scavenging activity.**

|  |  | TEAC of fractions | | Superoxide radical scavenging activity of  Fractions | | |
| --- | --- | --- | --- | --- | --- | --- |
| collected fractions | pooled fractions | N | mM TEAC / mg protein fractions | concentration (mg/ml) | N | % reduction ± standard deviation |
| A1-A7 | 1 | 3 | 0.97 ± 0.04 | 0.1 | 3 | 40.53 ± 2.26 |
| A8-A10 | 2 | 3 | 1.07 ± 0.02 | 0.1 | 3 | 27.16 ± 3.50 |
| A11-B6 | 3 | 3 | 1.10 ± 0.02 | 0.1 | 2 | ND |
| B7 | B7 | 3 | 0.64 ± 0.01 | 0.1 | 2 | ND |
| B8-C1 | 4 | 3 | 1.36 ± 0.13 | 0.1 | 2 | ND |
| C2-C9 | 5 | 2 | 1.22 ± 0.03 | 0.1 | 2 | ND |
| C10-D3 | 6 | 3 | 1.56 ± 0.16 | 0.1 | 2 | ND |
| D4-E2 | 7 | 2 | 2.19 ± 0.04 | 0.1 | 2 | ND |
| E3-E6 | 8 | 3 | 2.49 ± 0.00 | 0.1 | 2 | ND |
| E7-E11 | 9 | 3 | 18.20 ± 1.37 | 0.0125 | 2 | 64.15 ± 6.51 |
| E12-F6 | 10 | 3 | 0.98 ± 0.06 | 0.1 | 2 | ND |
| F7-G1 | 11 | 2 | 4.43 ± 0.21 | 0.05 | 2 | 79.09 ± 2.95 |
| G2-H1 | 12 | 2 | 2.45 ± 0.00 | 0.1 | 2 | ND |
| H2-H10 | 13 | 3 | 2.31 ± 0.03 | 0.1 | 2 | ND |
| H11-I9 | 14 | 2 | 2.18 ± 0.01 | 0.1 | 2 | ND |
| I10-J6 | 15 | 2 | 4.57 ± 0.02 | 0.05 | 2 | 86.46 ± 2.51 |
| J7-A’6 | 16 | 3 | 1.50 ± 0.02 | 0.1 | 2 | ND |
| A’7-C’1 | 17 | 4 | 4.38 ± 0.07 | 0.05 | 4 | 91.01 ± 1.69 |
| C’2-C’3 | 18 | 3 | No activity | - | - | - |

Note: N- number of experimental repeats, with each experiment done in triplets.

ND- not determined due to very low or nil activity.
