# Supplementary material for: Separation and identification of bioactive peptides from stem of Tinospora cordifolia (Willd.) Miers
Source: PLoS One. 2018 Mar 1;13(3):e0193717. doi: 10.1371/journal.pone.0193717 (PMC5832316; doi:10.1371/journal.pone.0193717)
Supplement: S6 Table — (DOCX) [file pone.0193717.s010.docx]

**S6 Table Sequence of five major peptides identified in**

**MALDI-MS spectra of fraction 9.**

| Molecular weight (Da) | Possible peptide sequence | De novo/MS BLAST Score |
| --- | --- | --- |
| 1678.76 | VLYSTPVKMWEPGR | 78.79 |
| 1450.71 | VITVVATAGSETMR | 78.52 |
| 1023.51 | HIGININSR | 81.18 |
| 1068.47 | KSSMETTIR | 66.95 |
| 1003.46 | ISHKYDIK | 84.09 |
